# Structure-Function of Neuronal Nicotinic Acetylcholine Receptor Inhibitors Derived From Natural Toxins

**DOI:** 10.3389/fnins.2020.609005

**Published:** 2020-11-25

**Authors:** Thao N. T. Ho, Nikita Abraham, Richard J. Lewis

**Affiliations:** Centre for Pain Research, Institute for Molecular Bioscience, The University of Queensland, St Lucia, QLD, Australia

**Keywords:** nAChRs, allosteric inhibitors, natural products, venom peptides, conotoxins, snake toxins, dinoflagellate toxins

## Abstract

Neuronal nicotinic acetylcholine receptors (nAChRs) are prototypical cation-selective, ligand-gated ion channels that mediate fast neurotransmission in the central and peripheral nervous systems. nAChRs are involved in a range of physiological and pathological functions and hence are important therapeutic targets. Their subunit homology and diverse pentameric assembly contribute to their challenging pharmacology and limit their drug development potential. Toxins produced by an extensive range of algae, plants and animals target nAChRs, with many proving pivotal in elucidating receptor pharmacology and biochemistry, as well as providing templates for structure-based drug design. The crystal structures of these toxins with diverse chemical profiles in complex with acetylcholine binding protein (AChBP), a soluble homolog of the extracellular ligand-binding domain of the nAChRs and more recently the extracellular domain of human α9 nAChRs, have been reported. These studies have shed light on the diverse molecular mechanisms of ligand-binding at neuronal nAChR subtypes and uncovered critical insights useful for rational drug design. This review provides a comprehensive overview and perspectives obtained from structure and function studies of diverse plant and animal toxins and their associated inhibitory mechanisms at neuronal nAChRs.

## Introduction

### Structure of Neuronal Nicotinic Acetylcholine Receptors (nAChRs)

**FIGURE 1:**
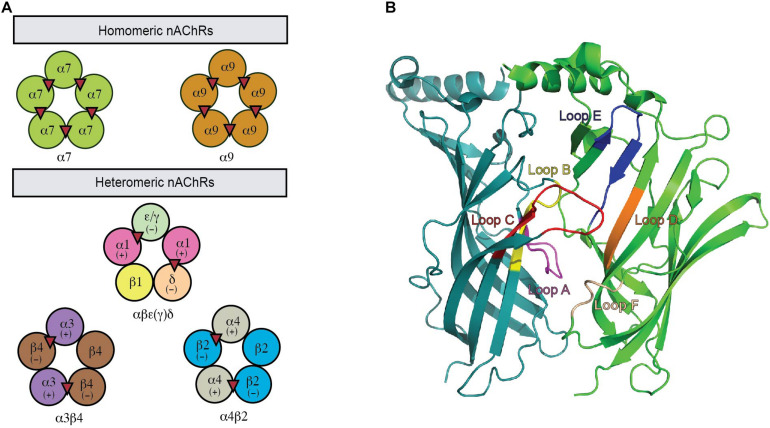
A functional nAChR is formed via the co-assembly of five subunits. **(A)** This pentameric complex can be homomeric or heteromeric combinations of α and β subunits. Acetylcholine binding sites are illustrated as red triangles. **(B)** The nAChR ligand-binding site is located between two adjacent protomers where the binding pocket is enclosed by loops (highlighted).

nAChRs are formed by the assembly of five transmembrane subunits. Seventeen different nAChR subunits have been identified so far in mammals, including ten α (α1–10), four β (β1–4), γ, δ, and ε subunits. Neuronal nAChRs are assembled either as homo-pentamers of α7, α8, and α9 or hetero-pentamers of α2–α6 in combination with β2–β4 or α9 with α10 subunits. In contrast, the hetero-pentameric muscle nAChRs comprise two α1 plus a β1, δ, and γ (fetal) or ε (adult) subunits (Figure 1A). The ligand binding pocket (LBP) for agonists or antagonist in nAChRs is at the interface between two neighboring subunits with one subunit being the principal face and the other being the complementary face (Figure 1B). In heteromeric nAChRs, the principal face comes from one α subunit, while the complementary face arises from non-α subunit. The binding of ligand stimulates different functional states of nAChRs via the conformational changes induced by the relative movement of the five subunits to each other (Liu et al., 2008). The structural characters of the LBP and the specific amino acid interactions between ligands and this site determine the conformational transitions that lie behind the pharmacological properties of a specific neuronal nAChR subtype (Dani and Bertrand, 2007). Thus, different pharmacological and biophysical properties are displayed by a diverse range of neuronal nAChR subtypes underpinned by the different subunit combinations. A complex expression profile in the nervous system is also exhibited by different subtypes of neuronal nAChRs. Together, this contributes to the complexity in the structure and function of neuronal nAChRs and their roles in the CNS.

### Pharmacology of nAChRs

nAChRs regulate the flow of mainly sodium, potassium and calcium ions across the cell membrane. The binding of ligands triggers a tertiary conformational transition of nAChRs among functionally distinct resting, open and desensitized states, with subunit composition and class of agonists influencing the kinetics of these conformational state transitions (Hurst et al., 2013). Agonists bound at the orthosteric site of nAChRs initially stabilize the open state and later a desensitized closed state, while effectors bound at the allosteric site can modify the energy barriers between transitions that shifts the equilibrium between states (Bertrand and Gopalakrishnan, 2007). Desensitization state may encompass short-and long-lived states of desensitization where the latter state is favored by long exposure to low concentration of agonists (Steinbach and Sine, 1987; De Biasi and Dani, 2011). Electrophysiology has been pivotal in determining the biophysical and pharmacological properties of different nAChRs subtypes. For example, the α7 nAChR is characterized by a low affinity for agonists, rapid activation, large conductance, high permeability to Ca^2+^ and fast desensitization, while α4β2 nAChRs and α3β4 nAChRs have slow inactivating nicotinic responses (Albuquerque et al., 2009). Interestingly, mutation of a single amino acid (L247T) in the ionic pore of chick α7 nAChRs caused pleiotropic effects on the nature of this receptor subtype, specifically the suppression of receptor desensitization, the increase in ligand affinity and the change in pharmacological profile of certain ligands from competitive antagonist into full agonists. These properties of this mutant are suggested to render a desensitized conductive state based on the basis of the allosteric model (Bertrand et al., 1992). This phenomenon has, in turn, shed light on the antagonism mechanism of certain antagonists from natural toxins, which are discussed later in this review.

### Therapeutic Implications of nAChRs

nAChRs are broadly distributed across the peripheral nervous system (PNS) and central nervous system (CNS) of both simple and complex organisms. This highlights the importance of nAChRs in the nervous system where they play a wide range of functions from the mediation of different cognitive processes to synaptic transmission from nerves to muscle. Homomeric α7 nAChRs and heteromeric α4β2^∗^ nAChRs are predominantly expressed in the human brain (Millar and Harkness, 2008; Colombo et al., 2013) where they contribute to the pathogenesis of a range of neurological disorders including Alzheimer’s disease, schizophrenia, Parkinson’s disease and depression (Freedman et al., 1995; Wang et al., 2000; D’Andrea and Nagele, 2006). α7 and α4β2 nAChRs also contribute to other non-neurological diseases, including a correlation of both subtypes with nicotine addiction and nicotine-induced behaviors (Buisson and Bertrand, 2002; Balfour, 2004) and the overexpression of α7 nAChRs associated with small-cell lung carcinomas (Sciamanna et al., 1997). Given their potential roles in disease development and progression, α7 and α4β2 nAChRs are currently one of the most studied nAChR subtypes. Recent studies are now starting to delineate roles for other nAChRs subtypes in a number of diseases. For example, despite the limited neuronal distribution of α6β2^∗^ subtypes, expression of the α6 subunit in nociceptors suggests it could contribute to sensory processing and pain (Hone and McIntosh, 2018), with an inverse correlation between CHRNA6 expression and neuropathic pain found in mice and humans (Wieskopf et al., 2015). More recently, the α9^∗^ has also been implicated in modulating the pathophysiology of neuropathic pain (Hone and McIntosh, 2018; Hone et al., 2018a). In contrast, dysfunction of muscle nAChRs results in the impaired neuromuscular transmission and muscle weakness typically associated with inherited mutations and acquired diseases such as myasthenia gravis or congenital myasthenic syndromes (Conti-Fine et al., 2006; Engel et al., 2015).

The therapeutically significant role of the nAChR subtypes in several pathophysiological conditions, together with the diversity in the subtype combinations, biophysical properties and expression patterns present a formidable challenge in rational drug discovery and design for this receptor family (Hogg et al., 2003b; Hogg and Bertrand, 2004). This urges for thorough insights into molecular and structural mechanisms governing nAChR subtype selectivity to facilitate successful therapeutic strategies for nAChR associated neuronal diseases (Lindstrom, 1997; Gotti and Clementi, 2004; Hogg and Bertrand, 2004).

### Tools to Study nAChR Structure

A breakthrough in characterization of nAChRs-ligand interactions came with the determination of the X-ray structure of acetylcholine binding protein (AChBP), a naturally occurring soluble protein homolog of nAChR (Brejc et al., 2001; Smit et al., 2001). Despite a low sequence similarity, AChBPs and nAChRs show remarkable structural homology (Brejc et al., 2001), including the orthosteric ligand recognition site formed by aromatic side chain residues found in nAChRs. However, the ligand-bound AChBPs still require the translation of information into individual nAChR subtypes via homology modeling in order to build a more accurate model for the interactions of ligands at targeted nAChRs.

A step forward in modeling the binding mechanism of ligands at nAChRs is to make AChBP resemble a given nAChR subtype. The crystal structure of the chimeric ligand binding domain of the human α7 AChR with AChBP was introduced via the substitution of selected native human α7 residues into *Lymnaea Stagnalis (Ls)* or *Aplysia californica* (*Ac)* AChBP (Li et al., 2011; Nemecz and Taylor, 2011). An alternative approach is the crystallization of an isolated component of the full length nAChR in complex with ligands at atomic level, which has been performed with neuronal nAChR α9 subunit extracellular domain (ECD). This approach could, in turn, improve the modeling of other neuronal nAChR ECDs (Dellisanti et al., 2007; Kouvatsos et al., 2016). Taken together, the co-crystal structure of nAChR structural surrogates (AChBP, chimera AChBP or nAChR ECD) in complex with different nAChR ligands is currently one of the most popular approaches for structure-function studies of nAChRs (Table 1). Importantly, inhibitors from natural toxins take up a high percentage of the co-crystal structures of ligands with nAChR structural surrogates.

**TABLE 1:** Co-crystal structure of naturally occurring nAChRs inhibitors with different AChBP.

Toxin	Compound	PDB	K_d_ (nM)				References
				
			*Ac*-AChBP	*Ls*-AChBP	nAChR subtype	Affinity (nM)	
Plant toxin	Methyllylcaconitine (MLA)	2BYR, 3SH1, 3SIO (α7/*Ls*-AChBP)	2.8	0.41	α7	0.025 (Chicken)	Palma et al., 1996; Yum et al., 1996; Hansen et al., 2002, 2004, 2005
	*d*-Tubocurarine (*d*-TC)	2XYT	509.2	170.7	α7	2,975 (Human)	Brams et al., 2011
	Strychnine	2XYS	38.0	223.5	α7	4,854 (Human)	Brams et al., 2011
	(+)-dihydro-β-erythroidine (DHβE)	4ALX	ND	52	α4β2	98 (Human)	Iturriaga-Vasquez et al., 2010; Shahsavar et al., 2012
Snake toxins	α-Cobratoxin (α-cbtx)	1YI5, 4D01 (α9 ECD)	191	3.2	α7	9 (α7–5HT_3_)	Fruchart-Gaillard et al., 2002; Hansen et al., 2004; Bourne et al., 2005; Zouridakis et al., 2014
	α-Bungarotoxin (α-bgtx)	3T4M (α7/*Ls*-AChBP)		27 (α7/Ls-AChBP)	α7	0.4 (Human)	Nemecz and Taylor, 2011
Conotoxins	PnIA[A10L D14K]	2BR8	32.6	27.5	α7	260 (Human)	Luo et al., 1999; Celie et al., 2005
	ImI	2C9T	33 (IC_50_)	4,140 (IC_50_)	α7	132 (Human)	Rogers et al., 2000; Ulens et al., 2006
	TxIA[A10L]	2UZ6	ND	6.2	α7	39 (Rat)	Dutertre et al., 2007
	GIC	5CO5	29 (IC_50_)	ND	α3β2	1.1 (Human)	Lin et al., 2016
	LsIA	5T90	5.44 (IC_50_)	210 (IC_50_)	α3β4	NA	Abraham et al., 2017
	LvIA	5XGL	131.6	ND	α4β2	46.8 (Human)	Xu et al., 2017
	PeIA	5JME	ND	ND	α6β4	9.9 (Human) 154 (Rat)	Hone et al., 2018b
	RgIA	6HY7	ND	ND	α9α10	1400 (Human)	Ren et al., 2019; Zouridakis et al., 2019
Phycotoxin	13-desmethyl spirolide C (SPX)	2WZY	0.019	1.2	α7	0.7 (Human)	Bourne et al., 2010; Hauser et al., 2012
	Gymonodimine A (GYM)	2 × 00	0.0047	0.0013	α7	1 (Human)	Bourne et al., 2010; Stivala et al., 2015
	Pinnatoxin A (PnTx-A)	4XHE	<0.05	170	α7	0.107 (Human)	Bourne et al., 2015
	Pinnatoxin G (PnTx-G)	4XK9	0.86	360	α7	5.06 (Human)	Bourne et al., 2015

## Natural Toxin Inhibitors at nAChRs

**FIGURE 2:**
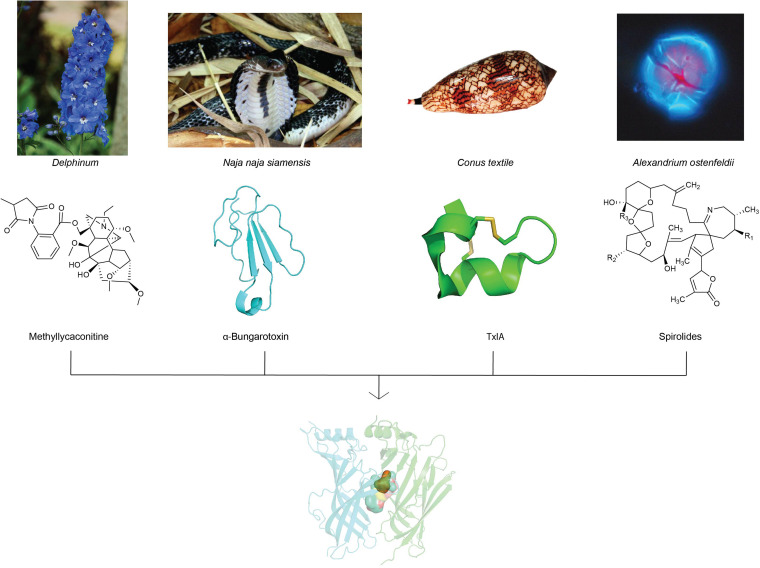
nAChR inhibitors isolated from toxins of plants, snake, cone snail and algae with distinct chemical profiles have been used extensively in structure-function studies to unravel the diverse molecular mechanisms of ligand-binding at neuronal nAChR subtypes. Images of representative source of toxins from plants (*Delphinum*), snake (*Naja naja siamensis*), cone snail (*Conus textile*), and algae (*Alexandrium ostenfeldii*) are displayed with their corresponding nAChR inhibitors chemical structures. The image of *Naja naja siamensissanke* is reprinted with permission from Dr. Jan Detka, Maj Institute of Pharmacology, Polish Academy of Sciences, Poland. The image of *Conus textile* is reprinted with permission for Dr. Himaya SWA, Institute for Molecular Bioscienc, Australia. The image of *Alexandrium ostenfeldii* is reprinted with permission from Bengt Karlson, SMHI, Sweden, source: Nordic Microalgae, http://nordicmicroalgae.org.

A wide variety of toxins from algae, plants, and animals target neuronal nAChRs to facilitate diverse prey capture and/or defensive strategies. In fact, naturally occurring toxins from snakes, plants, cone snails, and dinoflagellates dominate currently known nAChR antagonists (Daly, 2005) and have progressed our understanding of nAChR structure and function due to their often exquisite potency and selectivity. This review will focus on the chemistry and pharmacology of natural toxins inhibitors and the ligand-binding interactions fundamental in their antagonism at nAChRs (Figure 2).

### Snake Toxins

Snake venoms are comprised of a complex cocktail of proteins and peptides. These substances have optimally developed as lethal weapons for predation and defense against predators. Snake bite in humans can also have severe consequences including peripheral neurotoxicity, renal failure, severe necrosis at the bite site or coagulative and myotoxicity disorders that can be debilitating or even fatal (Fry et al., 2006; Zhang, 2015). One of the principal neurotoxic components of snake venom is a protein family termed three-finger toxins (TFTs). Discovered over forty years ago, TFTs remain valuable inhibitors for deciphering the molecular details of nAChRs, including the now famous α-cobratoxin (α-cbtx) isolated from *Naja naja siamensis* toxin and α-bungarotoxin (α-bgtx) from *Bungarus multicinctus* (Utkin, 2013) (Table 1).

#### Chemistry

**FIGURE 3:**
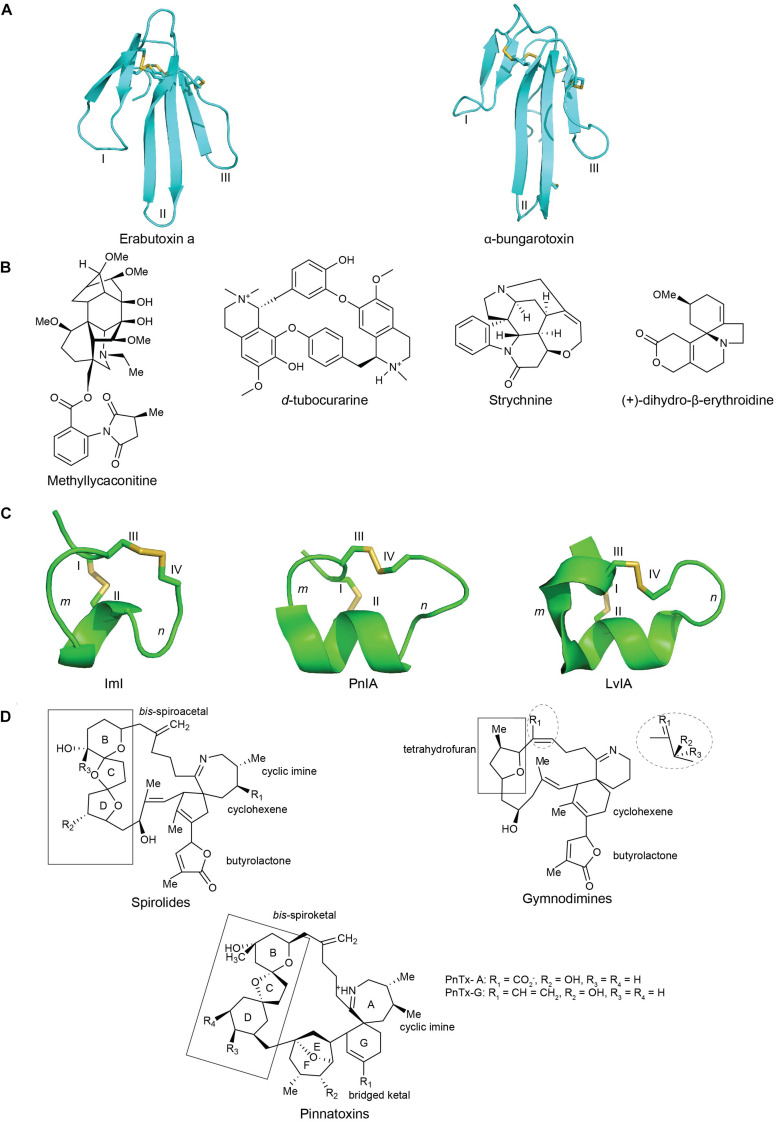
The structures of nAChRs inhibitors from plant, snake, cone snail and dinoflagellate toxins: **(A)** chemical structures of plant toxins: methyllylcacotinine, *d*-Tubocurarine, strychnine and (+)-dihydro-β-erythroidine (DHβE) (Daly, 2005); **(B)** three-dimensional structure of the three-finger snake toxins with three adjacent loop (I, II, and III): short chain α-neurotoxins erabutoxin a (PDB 5EBX) (Corfield et al., 1989) and long chain α-bungarotxin (PDB 1KFH) (Moise et al., 2002); **(C)** three-dimensional structures of α(*m/n*)-conotoxins with *m*, *n* being number of residues within the two loops formed by natively disulfide bond Cys^I^ -Cys^III^ and Cys^II^ -Cys^IV^ : ImI (PDB 1IMI) (Maslennikov et al., 1999), PnIA (PDB 1PEN) (Hu et al., 1996), and LvIA (PDB 2MDQ) (Luo et al., 2014); **(D)** chemical structures of spirolides, gymnodimines and pinnatoxins, reprinted from ref (Bourne et al., 2010; Otero et al., 2011; Bourne et al., 2015). Disulfide bonds in three-dimensional structures are colored in yellow.

TFTs are characterized by a distinct protein fold comprising of three adjacent β-stranded loops (fingers) emerging from a small, globular, hydrophobic core connected by four conserved disulfide bonds (Kessler et al., 2017). There are over five hundred TFTs discovered to date that encompass subtle variations in their loop sizes, turns and twists of various loops, and the number of β-strands. These features together contribute to their functional diversity (Dutertre et al., 2017). TFTs are classified into curaremimetic α-neurotoxins, κ-neurotoxins, and muscarinic toxins. α-Neurotoxins are further characterized into two major structural types: the short-chain α-neurotoxins with 60–62 amino acid residues and four disulfide bridges, and the long-chain toxins with 66–74 residues and five disulfide bonds (Tsetlin and Hucho, 2004; Dutertre et al., 2017; Figure 3A).

#### Pharmacology

The primary target of TFTs is the muscle-type nAChRs. Both the short and long-chain α-neurotoxins inhibit the skeletal muscle neuromuscular junctions at the same binding site with equal affinity. In fact, a breakthrough in nAChR research was facilitated by the discovery of the long-chain toxins α-bgtx (Chang and Lee, 1963). The high affinity binding of this α-neurotoxins to muscle-type nAChRs allowed the first isolation, identification and purification of nAChRs from the electric organ of *Torpedo marmorata* ray for biophysical characterizations (Karlsson et al., 1972; Olsen et al., 1972; Unwin, 1993; Utkin, 2013). However, a number of long-chain α-neurotoxins, including α-cbtx and α-bgtx, also inhibit neuronal α7 nAChRs with high affinity (Tsetlin and Hucho, 2004). Meanwhile, κ-neurotoxins preferably target neuronal α3-containing nAChRs (Chiappinelli, 1983; McLane et al., 1993).

### Plant Toxins

Molecules not required for normal plant physiology are termed as secondary metabolites (Green et al., 2013). These molecules exhibit diverse chemical structures ranging from the simple, low molecular weight molecules to the highly complex molecules, including toxins that perturb biological systems. Paralytic plant toxins have been used historically for hunting (Bisset, 1991) such as the *curares* that potently inhibit or activate nAChRs. Indeed, since their characterization, these toxins have been key tools to understanding nAChRs pharmacology (Daly, 2005). Among nAChR inhibitors from plants, a few notable plant toxins that have been studied extensively so far are methyllycaconitine (MLA) from *Aconitum and Delphinium (larkspur)* (Jennings et al., 1986), *d*-tubocurarine (*d*-TC) from *Chondrodendron tomentosium* plant (Wintersteiner and Dutcher, 1943) and strychnine from *Strychnos nux vomica* tree (Matsubayashi et al., 1998; Talcott, 2013) and (+)-dihydro-β-erythroidine (DHβE) from *Erythrina americana* species (Folkers and Major, 1937; Table 1).

#### Chemistry

Most plant toxin inhibitors, including DHβE, MLA, *d*-TC and strychnine, belong to the alkaloids group (Figure 3B). This class of naturally occurring organic compounds is characterized by their amino acid-derived nitrogen-containing bases (Rujjanawate et al., 2003). Alkaloids can be classified based on their structures, such as indoles, quinoline, isoquinolines, pyrrolidines, pyridines, pyrrolizidines, tropanes, and terpenoids and steroids (Hussain et al., 2018). MLA is a diterpenoid alkaloid possessing two main structural features responsible for its toxicity: an *N*-ethyl bicyclo tertiary alkaloid nitrogen atom and a C-18 anthranilic acid ester. Meanwhile, *d*-TC is a quinoline alkaloid (Kukel and Jennings, 1994) characterized by a monoquaternary monotertiary amine (Tuba et al., 2002). In addition, strychnine is a terpene indole alkaloid characteristic of a six-membered benzene ring fused to a five-membered nitrogen-containing pyrrole ring. This pyrrole ring with nitrogen atoms is responsible for the pharmacologically active properties of the indole ring (Rivera and Barrueto, 2014). On the other hand, alkaloids can also be categorized by its family of plant species. DHβE is a member of the *Erythrina* alkaloids having a unique tetracylic spiroamine scaffold. This scaffold allows DHβE to be a potential candidate to develop small subtype selective nAChR antagonists (Jepsen et al., 2014).

#### Pharmacology

Among the plant toxin inhibitors, MLA was first recognized for its insecticidal property arising from the potent antagonism of insect nAChRs (Jennings et al., 1986). Later, MLA was found to be a potent antagonist of α7 nAChRs with picomolar potency as evidenced from the block of ACh-induced currents in rat fetal hippocampal neurons (Ward et al., 1990). Its selectivity toward α7 nAChR was evidenced by its strong competition at the binding site of [^125^I]-α-bgtx in rat brain membrane and human muscle extract (Ward et al., 1990; Kukel and Jennings, 1994). *d*-TC antagonizes the muscle-type nAChRs as well as neuronal α7 nAChRs potently (Bertrand et al., 1990; Papineni and Pedersen, 1997). Its action as competitive neuromuscular blockers at the motor end plate underlies its pharmacological uses as muscle relaxants during surgeries (Sine, 2012). However, the intoxication of *d*-TC can result in complete paralysis of all skeletal muscles and fatality by respiratory paralysis. *d*-TC is also reported to target other member of the Cys-loop receptor (CLR) family such as glycine receptors or 5-hydroxytryptamine receptors (5-HT_3_) (Yakel and Jackson, 1988; Yan et al., 1998; Hope et al., 1999). Meanwhile, strychnine toxicity is reported to arise from its inhibition of glycine-gated Cl^–^ channels causing muscle spasm, convulsions and respiratory paralysis death (Johnson and Ascher, 1987) although potent antagonist at human α4β2 nAChRs and α7 nAChRs also contributes (Matsubayashi et al., 1998). Lastly, DHβE shows antagonistic preference toward α4β2 receptors as evidenced from its nanomolar affinity for α4β2 receptors compared to the micromolar potency exhibited at α7 and α3β4 nAChRs (Harvey and Luetje, 1996; Jensen et al., 2005; Iturriaga-Vasquez et al., 2010; Majinda, 2018).

### α-Conotoxins

Conesnails are marine gastropods of the genus *Conus* with around 700 species identified so far. Distinct sets of toxins have been developed by different *Conus* species as a survival strategy for feeding and defense (Lewis and Garcia, 2003; Lewis et al., 2012; Lebbe et al., 2014). This diverse mixture of biologically active compounds from *Conus* venoms has been optimally evolved as neurotoxins to target a broad range of ion channels with high potency and selectivity in the PNS and CNS (Lewis and Garcia, 2003; Lewis et al., 2012; Lebbe et al., 2014). In fact, α-conotoxins antagonizing nAChRs were one of the first classes of conopeptides discovered (McIntosh et al., 1999b; Dutertre et al., 2017). To date, α-conotoxins are among the best characterized conotoxins and the largest and most diverse groups of competitive antagonists at the orthosteric site of nAChRs.

#### Chemistry

α-Conotoxins belong to the A superfamily and are characterized by a CC-X_m_-C-X_n_-C cysteine framework, which allows for the formation of three possible disulfide connectivities: globular (I-III, II-IV), ribbon (I-IV, II-III) and bead (I-II, III-IV) (McIntosh et al., 1999a; Janes, 2005; Abraham et al., 2017) (Figure 3C). The globular conformation is generally the native bioactive isomer, while the ribbon and bead isomer typically show weak or no inhibition. Most α-conotoxins display a rigid and well-defined three-dimensional structure in solution due to the restraining disulfide bonds and a short 3_10_ α-helical backbone braced by the disulfide bond between Cys^I^ and Cys^III^ (Lewis and Garcia, 2003). α-Conotoxins are further divided into structural subgroups with different numbers of loop residues (*m/n*: 3/5, 5/5, 4/3, 4/4, 4/5, 4/6, and 4/7) between the disulfide bonds that roughly define their pharmacology. For example, the 3/5 framework α-conotoxins typically inhibit neuromuscular nAChRs, the 5/5, 4/3, 4/4, 4/5, and 4/6 subgroups mainly inhibit neuronal nAChRs, while the 4/7 subgroup can inhibit both neuronal and muscle subtypes. In addition, the first loop (*m*) consists of a conserved hydrophobic patch (Ser-Xaa-Pro), while the second loop (n) is typically more variable (Nicke et al., 2004; Lebbe et al., 2014; Dutertre et al., 2017). While additional cysteine frameworks have been identified more recently, the focus of this review are the typical α-conotoxins where co-crystal structures are available (Lewis et al., 2012).

#### Pharmacology

α-Conotoxins not only selectively block nAChRs but are also able to discriminate between the muscle and neuronal nAChRs subclasses. Remarkably, α-conotoxins can target different neuronal nAChRs subtypes with varying specificity despite their conserved globular fold (Lewis and Garcia, 2003). This makes α-conotoxins excellent tools for the differentiation of binding sites and the determination of ligand binding modes at distinct neuronal nAChR subtypes. Variations within the second loop of different α-conotoxins, even among α-conotoxins from the same subgroups, underlie this hypervariability in subtype selective pharmacology (Lewis et al., 2012). Additional factors, including C-terminal amidation, carboxylation, and sulfonation typically have a smaller influence of subtype selectivity (Ramilo et al., 1992; Craig et al., 1999; Nicke et al., 2003; Prashanth et al., 2012).

### Dinoflagellate Toxins

Cyclic imine toxins are lipophilic organic compounds found in marine micro-algae known as dinoflagellates. These toxins accumulate in bivalve molluscs through filter-feeding and produce adverse effects on human health (Picot et al., 2011). Several cyclic imine toxins have been well-studied, including 13-desmethyl spirolide C (SPX) from *Alexandrium ostenfeldii* and *Alexandrium peruvianum* (Cembella et al., 1999; Hu et al., 2001), gymonodimine A (GYM) from *Karenia selliformis* (Haywood et al., 2004) and pinnatoxin-A and G (PnTx-A and PnTx-G) from *Pinna attenuata* and *Pinna muricata* (Otero et al., 2011).

#### Chemistry

Cyclic imine toxins are macrocylic compounds containing an imine bond and spiro-linked ether moieties and include spirolides, gymnodimines, pinnatoxins, pteriatoxins, prorocentrolides, and spiro-proocentrimine (Figure 3D). Spirolides are the largest group of cyclic imines (Cembella et al., 1999; Hu et al., 2001) and are an economically important contaminant of shellfish. The structurally related pinnatoxins are amphoteric macrocyclic compounds that possess a 6,5,6- instead of the 5,5,6- *bis*-spiroketal found in spirolides as well as a bulky functionalized 5,6-bicycloketal ring (Otero et al., 2011). Lastly, gymnodimines contain a six-membered cyclic imine without methyl substituents, a trisubstituted tetrahydrofuran and an unsaturated lactone (Bourne et al., 2010) (Figure 3D). With its macrocyclic network, cyclic imines offer new avenues into the structural characterization of ligand binding mechanism at nAChRs.

#### Pharmacology

Cyclic imine toxins were first identified as fast-acting toxins that caused respiratory arrest in mouse bioassays (Munday et al., 2012; Stivala et al., 2015) associated with inhibition of both muscarinic and muscle-type α1_2_βγδ and neuronal α7, α4β2, and α3β2 AChRs (Hu et al., 2001; Bourne et al., 2010, 2015). Despite their potent neurotoxicity, cyclic imine toxins have not been well documented on their toxicological database, hence the lack of an acute reference dose. Thus, the amount of cyclic imines in shellfish is currently not regulated. However, at least regarding SPXs and GYMs, due to its high intraperitoneal toxicity in rodents, the limit level of these toxins is set based on the oral toxicity of laboratory animals (Molgó et al., 2017).

## Binding Interactions of Natural Toxin Inhibitors of nAChRs

### Overview of Structure-Function Studies of nAChRs

Understanding the structure-function relationship of nAChR ligands at atomic level was catalyzed by the determination of the X-ray structures of AChBPs (Brejc et al., 2001). This high-resolution structure facilitated the construction of accurate three-dimensional homology models of nAChRs allowing the construction of homology models and docking simulations to visualize the binding modes at nAChR antagonists. For example, the docking of α-cbtx at human α7 nAChRs illustrated that the toxin loop II positioned in the interface between two subunits and made extensive contacts with the C-loop (Fruchart-Gaillard et al., 2002). In comparison, α-conotoxins ImI, PnIA, PnIB, and MII docked at human α7 and α3β2 nAChRs models positioned at a small cavity located above the β9/β10 cleft with just few residues overlapping with the binding site of α-neurotoxins (Dutertre et al., 2004). The next major phase of structure-function studies was the co-crystal structures of nAChR with the nAChR agonists, nicotine and carbamylcholine. These agonists bound in a pocket formed by conserved aromatic residues from loop A, B, C, and D through cation-π interactions with the side chains of aromatic residues and a hydrogen bond between their polar nitrogen and the conserved Trp147 of loop B (Celie et al., 2004). Subsequently, nAChR antagonists were co-crystalized with AChBPs, revealing distinctive binding interfaces and conformations compared to those of agonists. One notable feature is “closed” loop C induced by agonists versus a more “open” form induced by antagonists. Importantly, these co-crystal structures (Bourne et al., 2005; Celie et al., 2005) confirmed the predictions from earlier docking studies, including the key pairwise interactions and the overlap of α-neurotoxins and α-conotoxins with the agonists binding site. A high number of nAChR agonists and antagonists have now been co-crystallized with AChBPs, greatly facilitating our understanding of the structure-function relationship of nAChR ligands. Among these co-crystal complexes, natural toxin inhibitors dominate, with their highly diverse chemical structures providing unique insight into the pair-wise interactions possible at nAChRs (Table 1). The following sections focus on the co-crystal structures of natural inhibitors in complex with nAChR structural surrogates to unravel the different binding modes underlying ligand interactions at nAChR.

### The Co-crystal Structure of Natural Toxin Inhibitors With AChBP

#### Snake Toxins

##### α-cbtx/Ls-AChBP

**FIGURE 4:**
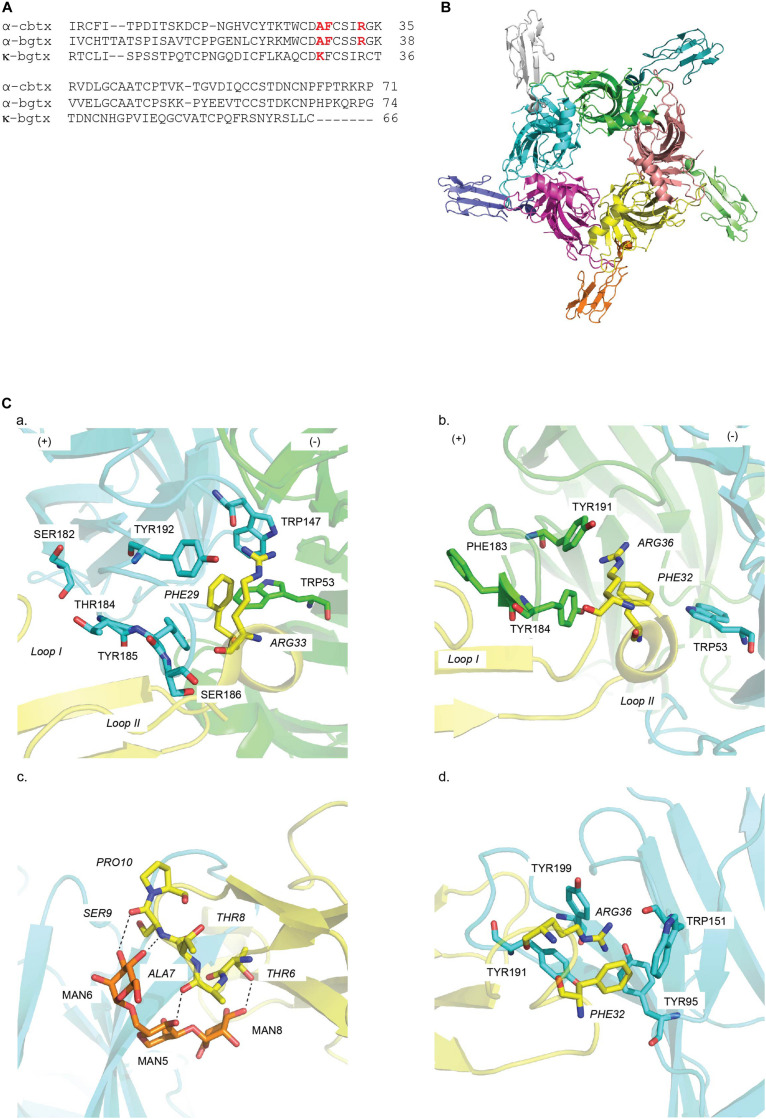
Structural mechanisms underlying snake toxin mediated nAChR inhibition: **(A)** Sequence alignment of snake toxins targeting nAChRs: α-cobratoxin (α-cbtx), α-Bungarotoxin (α-bgtx), and κ-Bungarotxin (κ-bgtx). Key residues in binding are highlighted in red. **(B)** α-cbtx occupied in all five binding pocket of *Ls*-AChBP as viewed along the AChBP fivefold axis (PDB 1YI5) (Bourne et al., 2005). **(C)** Key residues in the binding of **(a)** α-cbtx on the principal (+) (cyan) and the complementary (−) (green) face in *Ls*-AChBP (PDB 1YI5) (Unwin, 2005), **(b)** α-bgtx in the α7/Ls-AChBP chimera (Nemecz and Taylor, 2011) (PDB 3T4M), **(c)** mouse α1 nAChR subunit (PDB 2QC1) (Dellisanti et al., 2007) and **(d)** α9 nAChR ECD (PDB 4D01) (Zouridakis et al., 2014). Residues of snake toxins are italics. Hydrogen bonds are in dash line. MAN denotes for sugar moiety.

The α-cbtx/*Ls*-AChBP complex revealed for the first time the position and orientation of five TFTs binding at the interface of the five identical *Ls*-AChBP protomers (Figures 4A,B). The bound α-cbtx conformation determined by NMR was remarkably similar to its X-ray structure in complex with AChBP. Remarkably, the α-cbtx/*Ls*-AChBP complex reveals that the C-loop adopts a more open conformation compared to the previous co-crystal structures of AChBP with small molecule agonists. Hence, α-cbtx/*Ls*-AChBP crystal complex established for the first time the loop-C positioning has functional consequences. Further observations indicate that the tip of α-cbtx loop II lodges in the LBP with Phe29 localizing in the highly conserved aromatic residues of the principal side and Arg33 interacting against the complementary subunit (Figures 4A,Ca and Table 2). Notably, these two residues orient toward and partially overlap the nicotine binding site in AChBP, explaining their competitive interaction. These observations are also consistent with the previous docking studies of α-cbtx at the modeled human α7 nAChR derived from the crystal structure of AChBP (Fruchart-Gaillard et al., 2002). Meanwhile, the C-terminal region of α-cbtx and residues at the tip of α-cbtx loop III are solvent exposed and disordered, consistent with their weak contribution to binding. Finally, non-conserved residues in the C-loop of *Ls*-AChBP can interact with α-cbtx loop I (Figure 4Ca and Table 2) and are likely key selectivity determinants. Superimposing κ-neurotoxin and κ-bungarotoxin (κ-bgtx) into the α-cbtx/*Ls-*AChBP complex helped identify residues responsible for α3β2/α4β2 versus α7 nAChRs selectivity (Bourne et al., 2005). Specifically, these comparisons revealed that the shorter C-terminus of κ-bgtx makes extended contacts with the cationic Lys side chain of α3 and α4 subunits (equivalent to *Ls*-AChBP_Thr184) and allows a closer contact between κ-bgtx Lys29 at the tip of loop II and the complementary face of the β2 subunit.

**TABLE 2:** Molecular contacts between natural toxins and AChBP.

Toxins	Toxin contacts	AChBP contacts		References
		Principal side	Complementary side	
α-Cobratoxin (α-cbtx)	Phe29, Arg33	Trp143, Trp147, Tyr185, Tyr192,	Trp53	Bourne et al., 2005
	Loop I	Ser 182, Thr184, Ser186		
α-Bugarotoxin (α-bgtx)	Phe32, Arg36	Tyr184, Phe183, Tyr191*	Trp53	Nemecz and Taylor, 2011
	Phe32, Arg36	Tyr95, Tyr192, Trp151, Tyr199^#^		Zouridakis et al., 2014
MLA	*N-*phenyl succinimide	Ser94, Met126, Lys143, Gln186, Asp197	Gln38, Ser167	Hansen et al., 2005
	Ester linkage		Tyr55	
	*N-*ethylpiperidine ring	Trp147	Ile118	
	Lycoctonine	Trp147, Val148, Tyr188, Cys190, Cys191	Met116, Ile118	
*d*-Tubocurarine		Ser146, Trp147, Tyr188, Cys190, Cys191, Tyr193	Tyr55	Brams et al., 2011
Strychnine		Ser146, Trp147, Tyr188, Cys190, Cys191, Tyr193	Tyr55	Brams et al., 2011
DHβE		Tyr89, Trp143, Tyr185, Tyr192	Trp53	Shahsavar et al., 2012
PnIA[A10L D14K]	Leu5, Pro6, Pro7, Ala9, Leu10	Trp147, Tyr188, Tyr195		Celie et al., 2005
	Leu10	Val148	Val108, Met116, Ile118	
ImI	Arg7, Trp10	Asp75, Tyr195	Thr110	Ulens et al., 2006
TxIA [A10L]	Arg5	Tyr188, Asp197		Dutertre et al., 2007
	Leu10	Val148	Val108, Met116, Ile118	
GIC	His5	Tyr93, Tyr188, Tyr195		Lin et al., 2016
	Gln13		Thr110	
LsIA	Arg10		Gln55	Abraham et al., 2017
	Asn12		Gln74, Arg104	
PeIA	Pro13		Met116	Hone et al., 2018b
LvIA	Asn9		Gly32, Gln55, Met116	Xu et al., 2017
	Asp11	Gln153		
RgIA	Arg9	Trp151, Glu197, Pro200		Zouridakis et al., 2019
PnTx-A/PnTx-G	6,5,6-*bis*-spiroketal ring	Val148, Tyr188, Cys190, Cys191, Tyr195	Arg79, Val148	Bourne et al., 2015
	cyclic imine	Trp147		
	cyclohexene ring	Tyr93	Tyr55	
	carboxylate group	Tyr188	Ser167	
	5,6-bicycloketal substructure		Thr36, Ile118, Asp164, Ser166, Ser167	
13-desmethyl spirolide C (SPX)	*bis*-spiroacetal moiety	Tyr188, Cys190, Cys191, Tyr195	Val108	Bourne et al., 2010
	Cyclic imine	Trp147	Tyr93	
	Cyclohexene ring	Tyr93	Tyr55	
	(γ)-butyrolactone ring	Lys143, Tyr188		
Gymmonidimine A (GYM)	Tetrahydrofuran ring	Gln186, Cys190, Cys191, Tyr195	Val108, Val148	Hone et al., 2018a
	Cyclic imine	Trp147		
	Butylactone ring	Tyr93, Lys143, Tyr188	Tyr55	

* and ^#^ Residues on α7/Ls-AChBP chimera structure and α9-ECD, respectively.

##### α-bgtx/α7/AChBP

Another long-chain α-neurotoxin, α-bgtx, was crystallized with a chimera complex constructed from the human α7 nAChRs and AChBP (McLane et al., 1993; Huang et al., 2013) (Figures 4A,4Bc and Table 1). Similar features are shared between α-bgtx and α-cbtx when bound, specifically the toxin backbone orientation, the open C-loop conformation and the lodgement of C-loop between loop I and loop II of α-bgtx (Figure 4Cb). Despite that, these two complexes display a divergence in inter-residue interactions in which α-bgtx_Phe32 and Arg36, equivalent to α-cbtx_Phe29 and α-cbtx_Arg33, respectively, stack together and position in the aromatic cavity of the principal binding face (Figure 4Cb and Table 2). Particularly, Tyr184 coupling in in an energetical manner with its surrounding aromatic residues on the principal face underlie the activity of α-bgtx at targeted nAChR (Sine et al., 2013; Figures 4Bc,Cb). Evidently, the loss in α-bgtx affinity caused by the substitution of α7_C-loop into α-bgtx-insensitive α2/α3_C-loop was restored by the mutation of aromatic residues flanking Tyr184 on α2 or α3 subunit to their α7 counterparts. α-Bgtx was previously co-crystalised with mouse nAChR α1 subunit (α211). Superimposition of α-bgtx with α-cbtx binding to AChBP reveals comparable pairwise interactions (Dellisanti et al., 2007). Interestingly, finger I of α-bgtx makes polar contacts with the sugar mannose moiety of α211, a conserved feature in muscle nAChRs, suggesting the importance of sugars in the binding of α-neurotoxins to muscle nAChRs (Dellisanti et al., 2007) (Figure 4Cc and Table 2). Recently, α-bgtx has also been crystallized with human α9 nAChR ECD (Table 1). The overall binding mechanism shows high similarity to the α-bgtx/α7/AChBP complex despite the absence of the complementary subunit (Zouridakis et al., 2014) (Figure 4Cd). Finger II of α-bgtx also lodges against the principal side of α9 ECD with strong interactions with loop A, B, and C, while finger I and III display limited contacts (Figure 4Cd and Table 2). α-bgtx_Phe32 and Arg36 also reside in the aromatic pocket of α9 ECD as in the α7/AChBP chimera complex (Table 2). However, the lack of complementary subunit may cause α-bgtx to shift toward the binding site of α9 ECD by ∼4.5Å as compared to α7/AChBP chimera complex.

#### Plant Toxins

Like venom peptides, structures of plant inhibitors of the nAChRs in complex with AChBP reveal the plasticity of small molecular ligand interactions at the nAChR ligand-binding site of nAChRs.

##### MLA/Ac-AChBP Complex

**FIGURE 5:**
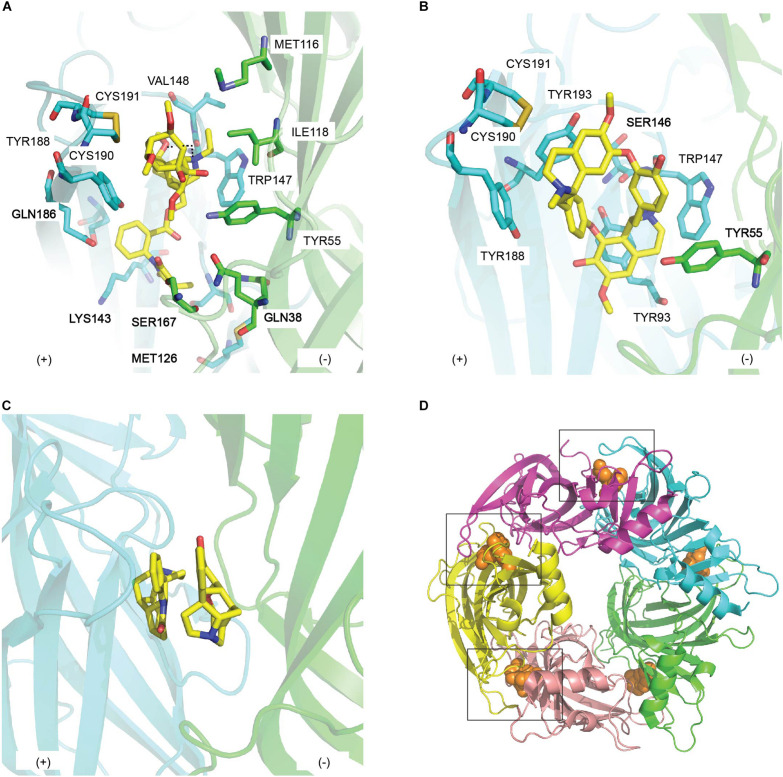
The binding mode of natural toxin inhibitors from plants at the principal (+) (cyan) and complementary (−) (green) face of AChBP. Key residues in the interactions between **(A)** methyllylcacotinine (PDB 2BYR) (Hansen et al., 2005) and **(B)** *d*-Tubocurarine (PDB 2XYT) (Brams et al., 2011) and AChBP; **(C)** the double occupancy mode displayed by strychnine in the co-crystal structure of strychnine/*Ac*-AChBP (PDB 2XYS) (Brams et al., 2011); **(D)** a close conformation of loop C induced by the binding of (+)-dihydro-β-erythroidine (PDB 4ALX) (orange sphere) in *Ls*-AChBP (Shahsavar et al., 2012).

The co-crystal structure of MLA and *Ac*-AChBP discloses the determinants of MLA binding to nAChRs. At the membrane side of LBP, *N-*ethylpiperidine ring in chair conformation stacks edge-to-face with Trp147. This orientation, in turn, positions the lycoctonine tertiary amine and the carbonyl oxygen of the ester linkage within hydrogen bonds with Trp147 of the principal face and Tyr53 of the complementary face, respectively. However, polar contact between the lycoctonine ring and *Ac*-AChBP is limited, hence the unchanged antagonism potency by simplified MLA derivatives lacking this ring (Bergmeier et al., 2004) (Figure 5A and Table 2). *N-phenyl* succinimide moiety displays extensive contacts in the LBP, consistent with the drop in MLA affinity on rat brain following ester hydrolysis to remove the N-*phenyl* succinimide moiety (Hansen et al., 2005) (Figure 5A and Tables 1, 2). Interestingly, MLA only induces an antagonist-bound “intermediate” movement of C-loop compared to the more open conformation seen with α-cbtx. Later, a similar binding orientation and conserved pairwise interactions to the MLA/*Ac*-AChBP are reported when MLA is co-crystallized with human α7/*Ac*-AChBP chimera (Nemecz and Taylor, 2011) and human α9 ECD (Zouridakis et al., 2014).

##### d-TC/Ac-AChBP

*d*-TC was co-crystallized with *Ac*-AChBP to understand its antagonism toward different members of CLR family (Brams et al., 2011) (Figure 5B). Interestingly, three different binding modes of *d*-TC are observed in the co-crystal structure of *d*-TC*/Ac*-AChBP, suggesting that *d*-TC can stabilize AChBP in a structurally distinct state. Despite this, most of the pairwise interactions between *d*-TC and the LBP are similar across the three binding modes where it makes contacts mainly with conserved aromatic residues in loop A, B (the principal side) and some residues on loop E (the complementary side) (Table 2). Some of these pairwise interactions confirm earlier computational model of *d*-TC bound to *Ls*-AChBP although the binding orientation is different. Particularly, α7_Ser148 of loop B and α7_Trp55 of loop D, equivalent to Ac-AChBP_Ser146 and *Ac*-AChBP_Tyr55, respectively, are identified as key determinants of *d*-TC potency as evidenced from a 148-fold and 14-fold reduction in *d*-TC potency following the alanine-scan mutagenesis on human α7 nAChRs (Figure 5B). Taken together, the interactions mostly with highly conserved residues in the binding pocket of different CLRs may underlie the low selectivity of *d*-TC.

##### Strychnine/Ac-AChBP

The broad specificity of strychnine was also investigated via its complex with *Ac*-AChBP (Brams et al., 2011). Compared to *d*-TC, four binding pockets are lodged by one strychnine with similar binding orientation, while the fifth binding pocket is occupied by two strychnine molecules in opposite orientations (Figure 5C). Despite this, strychnine and *d-*TC still show significant overlap in their pairwise interactions, particularly with the conserved amino acids in loop A, B, and D of the LBP (Table 2). Thus, a similar explanation as suggested for *d-*TC low selectivity could be also applied for strychnine. Remarkably, one of the residues found in the double strychnine occupancy mode in the crystal complex was previously characterized at α1 GlyR, implying the biologically relevance of double occupancy binding mode of strychnine (Grudzinska et al., 2005). A comparable analysis could be performed on human neuronal α7 nAChRs in order to examine whether this feature of strychnine is uniform across members of CLR family.

##### DHβE/Ls-AChBP

The crystal structure of DHβE/*Ls*-AChBP reveals the interacting surface of DHβE comprises a conserved aromatic pocket, identical to the co-crystal structures of other small molecules antagonists (Rucktooa et al., 2009; Shahsavar et al., 2012) (Figure 5D and Tables 1, 2). These observations also agree with alanine-scanning mutagenesis results for the equivalent residues on α4 (Harvey and Luetje, 1996). Interestingly, in the DHβE/*Ls*-AChBP complex the hydrogen-bonding network with the principal face and the water-mediated contacts with the complementary face are reminiscent of contacts seen in the complex of agonist like nicotine with AChBP (Celie et al., 2004). The C-loop of DHβE/*Ls*-AChBP is also in a closed orientation, identical to the C-loop conformation of nicotine/*Ls*-AChBP structure (Figure 5D). Previously, DHβE has been suggested to exert its inhibition at nAChRs by stabilizing the desensitized state instead of the resting state, given DHβE acts as an agonist at the mutated α7 [L247T] nAChR (Bertrand et al., 1992). Together, these features suggest that DHβE has a unique mode of antagonism at nAChRs compared to other prototypical antagonists.

#### α-Conotoxins

α-Conotoxins have been extensively used in structure-function studies of nAChRs as they offer broader nAChR subtypes selectivity including those that are less commonly targeted by natural product ligands.

##### PnIA [A10L D14K]/Ac-AChBP

**FIGURE 6:**
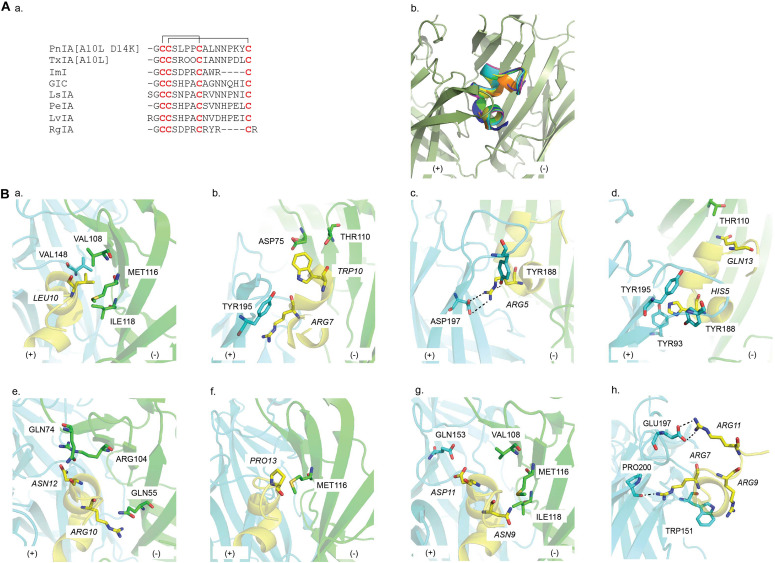
**(A) (a)** Sequence alignment of α-conotoxin targeting nAChRs; **(b)** a common binding mode at the interface of the principal (+) (cyan) and the complementary (−) (green) face in the AChBP binding pocket is presented by the overlay of the co-crystal structures of PnIA[A10L D14K], ImI, TxIA[A10L], GIC, LsIA, LvIA, and RgIA in AChBP. **(B)** Key residues in the binding of **(a)** PnIA[A10L D14K] (PDB 2BR8) (Celie et al., 2005), **(b)** ImI (PDB 2C9T) (Ulens et al., 2006), **(c)** TxIA[A10L] (PDB 2UZ6) (Dutertre et al., 2005), **(d)** GIC (PDB 5CO5) (Lin et al., 2016), **(e)** LsIA (5T90) (Abraham et al., 2017), **(f)** PeIA (PDB 5JME) (Hone et al., 2018b), **(g)** LvIA (PDB 5XGL) (Xu et al., 2017), and **(h)** RgIA (PDB 6HY7) (Zouridakis et al., 2019) at AChBP. Residues of α-conotoxin are italicized.

The PnIA [A10L D14K]/*Ac*-AChBP structure is the first reported α-conotoxin co-crystal complex (Celie et al., 2005) (Table 1). PnIA from *Conus pennaceus* competitively inhibits α3β2 nAChRs with substantially higher affinity than α7 nAChRs (Fainzilber et al., 1994) (Figure 6Aa). However, A10L mutation shifted PnIA selectivity from α3β2 nAChRs toward α7 nAChRs and D14K mutation further enhanced PnIA[A10L] efficacy by threefold at α7 nAChRs (Hogg et al., 1999; Luo et al., 1999; Celie et al., 2005) (Table 1). The PnIA [A10L D14K]/*Ac*-AChBP establishes the general binding mode of α-conotoxins at LBP, in which the α-conotoxin N-terminal and C-terminal orient toward the membrane side and the top of the receptor, respectively, while the α-helical backbone is buried in the aromatic cage (Figure 6Ab). The characteristic stacking of α-conotoxin Cys^I^ -Cys^III^ bond onto the vicinal Cys190-Cys191 disulfide bond of AChBP is also first described. Further observation reveals that PnIA [A10L D14K] shares an identical stabilizing movement of loop C to α-cbtx despite the huge difference in size and chemical properties. However, compared to α-cbtx, PnIA [A10L D14K] is buried deeper in the LBP and interacts with multiple residues on both faces of the LBP. The observed pairwise interactions here are in agreement with the earlier α7 nAChRs mutagenesis study (Quiram et al., 1999). Particularly, the key role of Leu10 in conferring PnIA specificity and affinity for α7 nAChR arises from its position within the hydrophobic pocket comprising of Val148 (principal side), Val108, Met116, and Ile118 (complementary side) (Figure 6Ba and Table 2). Interestingly, PnIA inhibits the non-desensitizing α7 [L247T] nAChRs, while PnIA [A10L] and PnIA [A10L D14K] activate this mutant receptor (Hogg et al., 2003a). Thus, despite the similar conformation of the C-loop, this agonist feature of PnIA [A10L D14K] at α7 [L247T], together with no obvious changes in interface loops, the PnIA [A10L D14K]/*Ac*-AChBP structure was suggested to be in a desensitized state instead of a resting state (Celie et al., 2005).

##### ImI/Ac-AChBP

The small ImI from *Conus imperialis* is a potent α7/α3β2 nAChRs blocker (McIntosh et al., 1994) (Figure 6Aa and Table 1). However, ImI is mostly studied for its potency toward α7 nAChRs. In the ImI/*Ac*-AChBP complex, an identical binding position and orientation to PnIA[A10L D14K] are exhibited (Ulens et al., 2006) (Figure 6Bb). Both PnIA[A10L D14K] and ImI share the same stacking between Cys^I^ -Cys^III^ bond and the vicinal disulfide bond Cys190-Cys191. Yet, ImI presents with a broader range of interactions that only partially overlaps with those seen in the complex of PnIA[A10L D14K]. Specifically, the side chains of ImI Arg7 and Trp10 protrude deep into the binding site and interact with both the principal and complementary side (Figure 6Bb and Table 2). These observations are supported by the earlier mutagenesis data of ImI on α7 nAChR, revealing vital roles for Arg7 and Trp10 in ImI for high affinity interactions at α7 nAChR (Quiram et al., 1999). Similar to PnIA [A10L] and PnIA [A10L D14K], ImI evokedcurrents at α7 [L247T] nAChRs and the interface loops of the ImI/*Ac*-AChBP also showed no changes in its interface conformations as seen in the PnIA [A10L D14K]/*Ac*-AChBP structure. Thus, ImI is proposed to stabilize the ligand binding domain in a desensitized conformation (Ulens et al., 2006).

##### TxIA/Ac-AChBP

TxIA from *Conus textile* was the first α-conotoxin isolated by assay guided fractionation using AChBP (Dutertre et al., 2007) (Figure 6Aa and Table 1). Similar to PnIA[A10L], the substitution of Ala into Leu at position 10 of TxIA also enhances its potency at α7 nAChR. As expected, the TxIA[A10L]/*Ac*-AChBP structure reveals the position of TxIA [A10L]_Leu10 in the same hydrophobic pocket as in the PnIA[A10L D14K]/*Ac*-AChBP complex (Figure 6Bc). This phenomenon reinforces the importance of a long-chain hydrophobic residue at position 10 in conferring α-conotoxin selectivity toward α7 nAChRs. However, compared to PnIA[A10L D14K] and ImI, a 20° backbone tilt downward around Pro7 of TxIA[A10L] is observed, which allows Arg5 to form a hydrogen with Tyr188 and a salt bridge with Asp197 deeper in the α-conotoxin binding pocket (Figure 6Bc). Although Asp197 is conserved among *Ac*-AChBP, β2 and α7 subunit, its interaction with TxIA_Arg5 was only observed experimentally on α3β2 nAChRs but not on α7 nAChRs. This suggests that TxIA[A10L] may exhibit a different binding conformation at α7 nAChRs compared to α3β2 nAChRs that shifts the importance of the salt bridge between TxIA[A10L]_Arg5 and Asp197 (Dutertre et al., 2007).

##### GIC/Ac-AChBP

GIC from *Conus geographus* inhibits neuronal α3β2 nAChRs at low nM concentration (McIntosh et al., 2002; Lin et al., 2016) (Figure 6Aa and Table 1). From the complex of GIC/*Ac*-AChBP and the homology model of GIC bound to different nAChR subtypes, His5 and Gln13 are identified as key residues in the activity and selectivity of GIC at α3β2 nAChRs, respectively (Figure 6Bd). His5 shows extensive contacts with the conserved aromatic binding pocket and as expected, a significant drop in GIC at both *Ac*-AChBP and α3β2 nAChR was reported following the substitution of His5 into Ala (Figure 6Bd and Table 2). A favorable interaction between GIC_Gln13 and *Ac*-AChBP_Thr110 is described. As the equivalent residues of *Ac*-AChBP_Thr110 on β2 and β4 are Ser113 and Arg155, respectively, this interaction could remain on β2, while a steric clash could be formed on β4 interface.

##### LsIA/Ls-AChBP

LsIA from *Conus limpusi* is an equipotent blocker of human α7 and rat α3β2 nAChRs but is inactive at α3β4 nAChRs despite a high sequence similarity with other α3β4-active α-conotoxins (Inserra et al., 2013) (Figure 6Aa and Table 1). To characterize the pharmacophore governing α-conotoxin antagonism at α3β4 nAChRs, Abraham et al. (2017) generated a co-crystal structure of LsIA/*Ls*-AChBP and docking models at α3β4. These studies reveals that Arg10 has an electrostatic clash with the positively charged β4_Lys61 (Figure 6Be and Table 2) that was removed when Arg10 was replaced with uncharged side chain residues to enhance activity at α3β4 nAChRs. Similarly, the mutation of *Ls*-AChBP_Gln55 into the equivalent residue on β4_Lys61 caused a 100-fold drop in LsIA potency. Additionally, Asn12 in the conserved -NN- motif for the first time was found to form hydrophobic interactions with Ile81 and Ile113 of the β4 complementary face, while it exhibits polar contacts with the equivalent residues on *Ls*-AChBP (Gln74 and Arg104) and α7 (Thr75 and Leu107) (Figure 6Be and Table 2). As expected, enhancing the hydrophobic interactions at the β4 subunit increased LsIA potency at α3β4 nAChRs but reduced potency at α7 nAChRs, with the double mutant LsIA [R10F N12L] exhibiting > 250-fold selectivity toward α3β4 over α7 activity. Thus, interactions with the triad composing of Lys61, Ile81, and Ile113 are proposed to be key contacts for the antagonism of α-conotoxins at α3β4 nAChRs.

##### PeIA/Ac-AChBP

PeIA from *Conus pergrandis* is a potential candidate for the development of treatment for pain-related conditions due to its potency at α6^∗^ nAChR (Hone et al., 2013) (Figure 6Aa and Table 1). However, one drawback in the evaluation of its potential for modulating pain clinically is the discrepancy in the ligand sensitivity between receptors in human and rodent models (Satkunanathan et al., 2005; Hone et al., 2018b). Sequence alignment of human and rat α6 subunit revealed that residues forming the ligand-biding pocket are mostly conserved between the two species except for a Leu-Gln difference at position 119. The co-crystal structure of PeIA/*Ac*-AChBP presents a close contact between Pro13 and *Ac*-AChBP_Met116, equivalent to human β4_Leu119 and rat β4_Gln119 (Figure 6Bf). Site-directed mutagenesis studies and structure-activity studies confirmed these observations, with human β4 _Leu119 being responsible for PeIA higher sensitivity at human α6/α3β4 nAChRs and PeIA_Pro13 being critical for PeIA high potency (Hone et al., 2018b). Interestingly, the same mutation of PeIA_Pro13 resulted in differential sensitivities of PeIA on human versus rat α6/α3β4 nAChRs. This result implies that the LBP of human and rat α6/α3β4 nAChRs differ despite the high similarity in their ligand-binding domain sequence, likely reflecting a tight lock-and-key binding mode, which needs to be considered before extrapolating results on α-conotoxin-nAChR interactions between species.

##### LvIA/Ac-AChBP

α-Conotoxin LvIA, cloned from *Conus lividus* genomic DNA, exhibits a high preference for α3β2 over α6β2^∗^ nAChR despite similarities in α3 and α6 subunit sequences (Luo et al., 2014) (Figure 6Aa and Table 1). As observed in homology models built from the co-crystal structure of LvIA/*Ac*-AChBP, while Asp11 forms a salt bridge with rat α3_Lys155 (equivalent to *Ac*-AChBP_Gln153), it displays an electrostatically repulsion with rat α6_Glu155 (Figure 6Bg and Table 2). This contact could underlie the LvIA preference toward α3^∗^ over α6^∗^ subunit. Additionally, the localization of Asn9 in a hydrophobic pocket comprising of Met36, Thr59, and Phe119 in α3β2 model (equivalent to Gly32, Gln55, and Met116 of *Ac*-AChBP) is proposed to further account for LvIA selectivity toward α3β2 nAChRs (Figure 6Bg and Table 2). Thus, Asp11 and Asn9 are identified as key determinants in the high potency of LvIA at α3β2 nAChRs compared to other nAChR subtypes.

##### RgIA/α9-ECD

RgIA from *Conus regius* specifically targets α9α10 nAChRs (Ellison et al., 2006, 2008) (Figure 6Aa and Table 1). RgIA is the first α-conotoxin to be co-crystalised with nAChR ECD (Zouridakis et al., 2019). Overall, the superimposition between the RgIA/α9-ECD complex and other α-conotoxin complexes shows a high similarity, validating the usefulness of using the principal side of α9-ECD for structural studies (Ulens et al., 2006). The Asp-Pro-Arg triad of RgIA as well as Arg11 is observed to be involved in a number of interactions between RgIA and α9-ECD, which agrees with previous mutational studies looking at RgIA activity on α9α10 nAChRs (Ellison et al., 2006, 2008) (Figure 6Bf and Table 2). Among the three possible putative binding sites of α9α10 nAChRs, namely the α9(+)/α9(−), α9(+)/α10(−), and α10(+)/α9(−), MD simulations constructed from the co-crystal structure suggest that RgIA prefers to bind at the binding interface formed by either α9 or α10 as the principal side and α9 as the adjacent complementary side, rather than α10. This complex has given valuable insight on the possible stoichiometry of this subtype, which may be useful for the design of RgIA analogs targeting human α9α10 nAChR.

#### Phycotoxins—Cyclic Imines

##### SPX/Ac-ChBP

**FIGURE 7:**
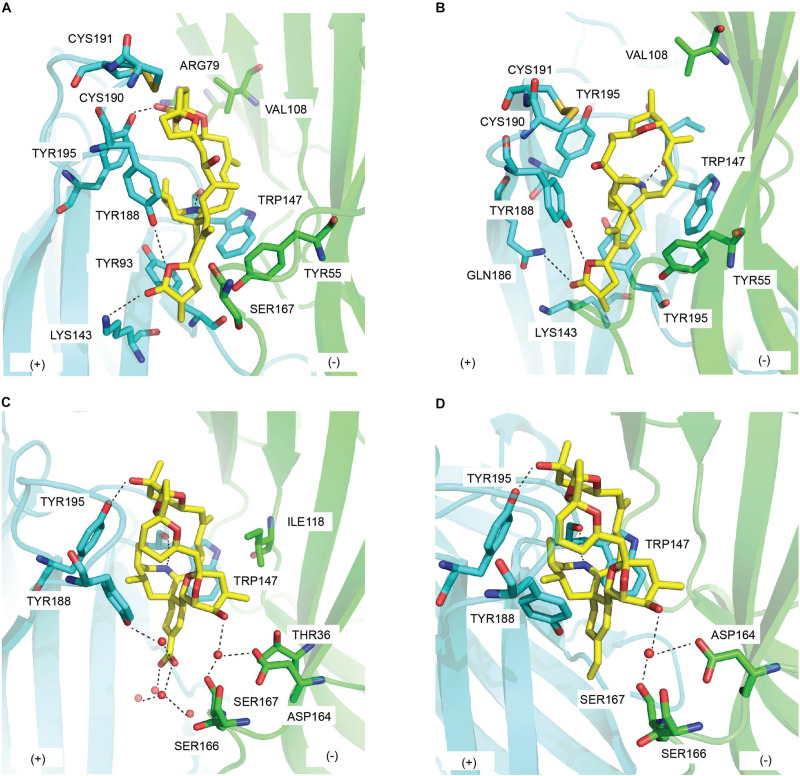
Key residues in the binding of cyclic imine toxins from dinoflagellate at the principal (+) (cyan) and the complementary (−) (green) face of AChBP: **(A)** 13-desmethyl spirolide C (PDB 2WZY), **(B)** gymonodimine A (PDB 2X00) (Bourne et al., 2010), **(C)** pinnatoxin-A (PDB 4XHE), and **(D)** pinnatoxin G (PDB 4XK9) (Bourne et al., 2015). Hydrogen bonds are in dash line.

In the co-crystal structure with *Ac*-AChBP, SPX spans the long axis of *Ac*-AChBP with *bis*-spiroacetal ring at the apical face and the (γ)-butyrolactone moiety at the membrane face (Bourne et al., 2010) (Figure 7A and Tables 1, 2). The carbon skeleton of SPX behaves similarly to the bulky oxygen-rich lycaconitine skeleton of MLA, hence the high resemblance between the C-loop movement of SPX/*Ac*-AChBP complex and that of MLA complex. However, the *bis*-spiroacetal ring system of SPX that is absent in MLA could be responsible for the 600-fold higher potency of SPX at *Ac*-AChBP and *Ls*-AChBP compared to MLA due to its involvement in multiple interactions with the C-loop (Table 1). Notably, the hydroxyl and methyl substitutions of the tetrahydropyran ring (ring B) form hydrogen bond with Tyr195 of the principal side and interact with Val108 of the complementary side, which could underlie the higher potency of SPX at α7 nAChRs than at α4β2 nAChRs (Figure 7A). SPX could be destabilized in the β2 binding pocket due to the less bulky hydrophobic residue β2_Val108 compared to α7_Leu108 (equivalent to *Ac*-AChBP_Val108). This causes the spiroacetal moiety to reposition in α4β2 nAChRs, resulting in the loss of the hydrogen bond between this moiety and Tyr195 (Aráoz et al., 2015). In addition, the imine ring acts as a hinge point for SPX via a number of hydrogen bonds at the LBP, but only shows sparing contacts with loop F on the complementary face. In the membrane side, only weak hydrogen bonds with *Ac*-AChBP are formed by terminal (γ)-butyrolactone ring of SPX.

##### GYM/Ac-ChBP

A well conserved interacting network is observed between the co-crystal structure of GYM/*Ac*-AChBP and that of SPX (Bourne et al., 2010) (Figure 7B and Tables 1, 2). The tetrahydrofuran ring system of GYM, equivalent to the *bis*-spiroacetal ring of SPX, displays similar interactions with conserved residues in the LBP. The central cyclic imine and the (γ)-butyrolactone ring of GYM share comparable contacting surface with SPX. Particularly, a limited interaction with loop F residues is also exhibited by GYM. However, due to the smaller size of tetrahydrofuran ring compared to the *bis*-spiroacetal ring, GYM is wrapped by the Gln186-Tyr195 segment of loop-C to a further extend than SPX and adopts a flat conformation with more flexibility within the binding pocket. This C-loop movement of GYM introduces further van der Waals with Tyr93 of the principal side (Figure 7B).

##### PnTxs/Ac-AChBP

The overall orientation of PnTx-A and PnTx-G in the crystal structure similarly expands from the apical face along to the membrane face and complements the shape of the LBP with multiple interactions (Bourne et al., 2015) (Figures 7C,D and Tables 1, 2). A hydrogen bond with Ser167 (loop F) and a number of water-mediated bridges with Tyr188 (loop C) are formed between (name the nAChR) and the carboxylate group (R_1_ substituent) of ring G in PnTx-A, while the less polar vinyl group in PnTx-G shows weak interactions with Ser167. The more muscle-selective PnTx-G could be explained by these interactions between R_1_ substituent of ring G with LBP as evidenced by a reduction in *in vitro* neuromuscular blocking activity and *in vivo* toxicity exhibited by a PnTx-F derivative with fluorophore label in substitution for R_1_ substituent of ring G (Hellyer et al., 2014). In addition, the exocyclic hydroxyl (R_2_ substituent) of the unique bulky bridged 5,6-bicycloketal substructure (ring EF) of PnTx-A and PnTx-G exhibits extensive contacts with Asp164 and the neighboring Ser166-Ser167 on loop F as well as with Thr36 in strand β1 and Ile118 on loop E (Figures 7C,D and Table 2). These interactions with loop F are proposed to be one of the key determinants in the higher affinity of PnTxs for *Ac*-AChBP compared to *Ls*-AChBP as well as the selectivity of PnTxs toward neuronal α7 nAChRs (Table 1). To be specific, the exocyclic methyl and hydroxyl groups in ring EF could sterically clash with the bulkier Tyr164 of loop F and Lys35 of strand β1 on *Ls*-AChBP (equivalent to Ser167 and Thr36 on *Ac*-AChBP, respectively). Similarly, both PnTx-A and PnTx-G may form unfavorable interactions with charged β2_Asp that is equivalent to *Ac*-AChBP_Ser167 and α7_Gly167.

### Perspectives

#### Comparison Between Binding Modes of Natural Toxin Inhibitors and Endogenous Agonists

**FIGURE 8:**
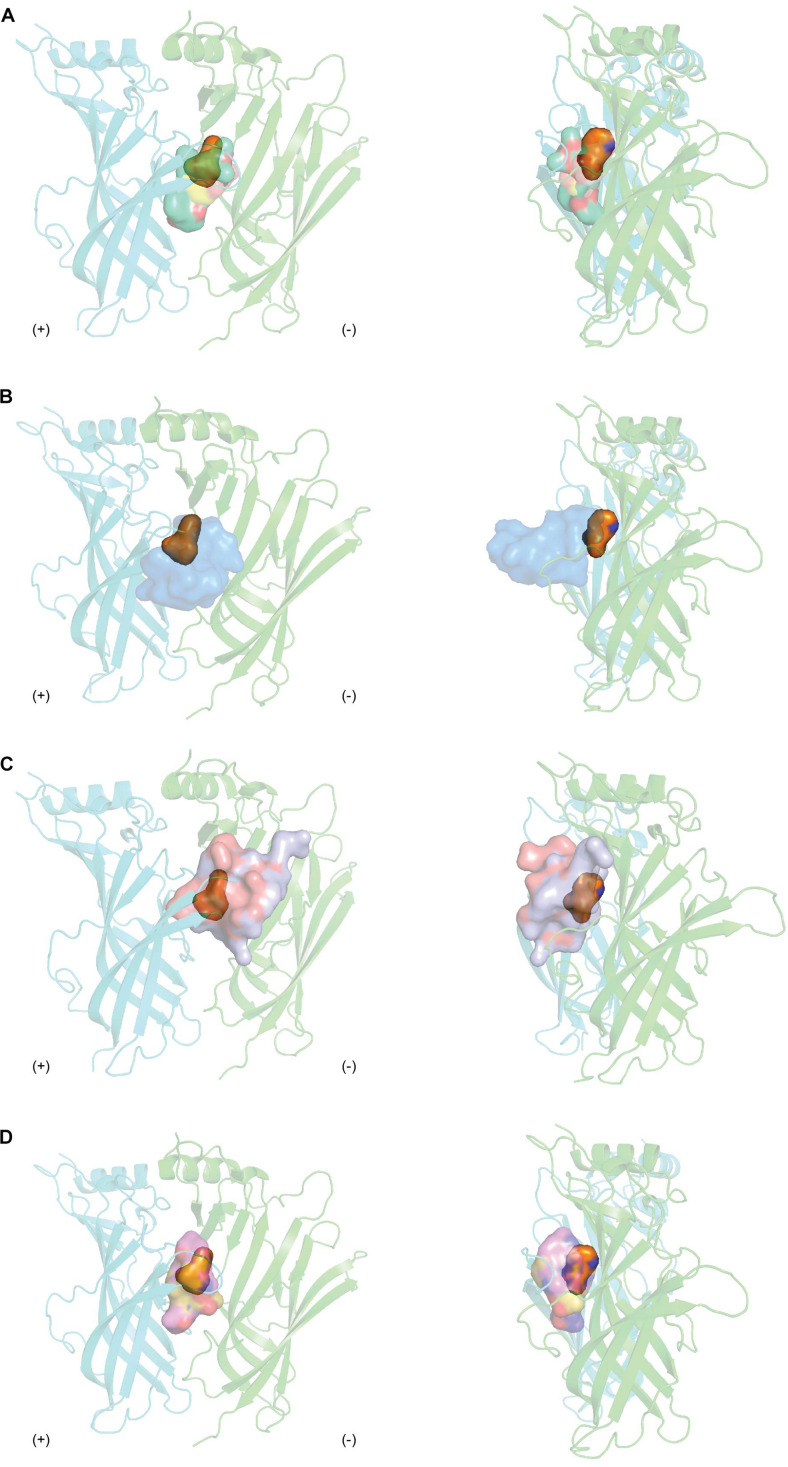
The overlay binding positions of nAChR inhibitors from natural toxins (surface in transparency) with nAChR agonist nicotine (orange surface in transparency) (PDB 1UW6) (Celie et al., 2004) at the protomer-protomer interfaces formed by the principal (+) (cyan) and complementary (−) (green) side of AChBP as viewed from front (right panel) and from side (left panel). **(A)** The overlayed binding surfaces of methyllylcacotinine (PDB 2BYR) (Hansen et al., 2005), *d*-Tubocurarine (PDB 2XYT) (Brams et al., 2011), strychnine (PDB 2XYS) (Brams et al., 2011) and (+)-dihydro-β-erythroidine (PDB 2XYS) (Shahsavar et al., 2012) show overlapping binding region with that of nicotine. **(B)** The tip of α-cbtx loop II inserts in the binding interface and orients toward the partially overlapping regions where nicotine binds (PDB 1YI5) (Bourne et al., 2005). **(C)** The binding regions of α-conotoxins (PnIA[A10L D14K] (PDB 2BR8) (Celie et al., 2005) and ImI (PDB 2C9T) (Ulens et al., 2006) cover the apical and central surfaces of the binding pocket as well as extend toward loop F. **(D)** The macrocyclic framework of cyclic imines, including 13-desmethyl spirolide C (PDB 2WZY), gymonodimine A (PDB 2X00) (Bourne et al., 2010), and pinnatoxin-A (PDB 4XHE) (Bourne et al., 2015) radially complements the binding pocket.

Agonists are positioned in a core motif formed by conserved aromatic residues central to the LBP as observed from the co-crystal structures of agonists like nicotine or acetylcholine in complex with AChBP (Celie et al., 2004; Olsen et al., 2014). The binding of agonists to AChBP induces states resembling either the activated or the subsequently desensitized state of the nAChR, with a closed C loop conformation. Unfortunately, given the extent nAChR subtypes influence the rates receptors open and then desensitized, it remains challenging to distinguish between these states in AChBP (Giniatullin et al., 2005). Meanwhile, competitive antagonists exert its pharmacological characteristic by positioning itself in the overlapping regions of agonists binding. Antagonists from natural toxins, regardless of their distinct chemical structures, cover a more extended area in the LBP from apical to membrane side (Figure 8). This binding mechanism results in the opening of C-loop on the principal face, resembling the EM structure of nAChR in the resting state (Unwin, 2005). Thus, antagonists are proposed to lock the C-loop in a resting state that obstructs the ligand-binding site and prevents the initiation of the signal inducing channel opening. This is consistent with the phenomenon that competitive antagonists such as MLA or α-bgtx remain agonists at α7 [L247T] and α-bgtx binding affinity is unchanged at this mutated receptor. In contrast, PnIA [A10L], PnIA [A10L D14K], ImI and DHβE are suggested to inhibit nAChRs by stabilizing the desensitized state rather than the non-activated state of the receptor, given α7 [L247T] nAChR transforms these to agonists (Bertrand et al., 1992; Hogg et al., 2003a; Celie et al., 2005; Ulens et al., 2006). Despite the outward movement of C-loop observed in their co-crystal structures, no changes in the conformation of the interface loops that could distinguish between the resting and activated state were reported. These features suggest either that C-loop movement may not be coupled to the interface loops or that AChBP could be trapped in a conformation similar to the desensitized state. In contrast, DHβE induces closure of the C-loop and a hydrogen bonding network similar to that of agonists (Shahsavar et al., 2012), suggesting a different mechanism of antagonism by DHβE compared to prototypical competitive antagonists. Additionally, major positional changes of loop F on the complementary face are also induced upon antagonists binding, potentially underlying the greater subtype selectivity of natural toxin antagonists compared to agonists.

#### Comparison Among Binding Modes of Natural Toxin Inhibitors

The diverse chemical profiles of natural nAChRs inhibitors from different sources give multifaceted insights in the ligand-interaction mechanisms at nAChRs. The high potency and high selectivity at nAChR subtypes shared by MLA, α-neurotoxins, α-conotoxins and cyclic imine toxins can be ascribed to their highly specific pairwise interactions highlighted in their co-crystal structures with AChBP. MLA complements the ligand binding pocket precisely via three main substructures acting as hinge regions. α-Neurotoxins and α-conotoxins residues show exclusive contacts in the LBP. Meanwhile, PnTxs extend from the membrane to the apical regions of the AChBP which allows the donation of hydrogen bond by the functional imine group as well as the complementation of other functional groups to the LBP.

On the other hand, the low selectivity exhibited by some toxins like *d*-TC, strychnine, SPX, and GYM could stem from their limited diversity in interactions at the LBP. The interacting surface of *d*-TC and strychnine constitute mainly of highly conserved residues among different members of CLRs. This is in contrast to the broader range of contacts displayed by those aforementioned nAChRs-selective toxins. Moreover, the extent of loop F involvement in the ligand-binding interactions could also further contribute to toxin low selectivity at different neuronal nAChR subtypes as well as between muscle and neuronal nAChRs. As described above, only a few interactions are seen between SPX and GYM with loop F where most of the non-conserved residues among nAChR subunits are located. Meanwhile, peptidic toxins like α-conotoxins display multiple contacts with residues on loop F. These observations are indeed consistent with the suggestion that loop F is responsible for determining nAChR subtype specificity (Bourne et al., 2015).

Unusual binding mechanisms are exhibited by some traditional nAChR inhibitors as observed in their co-crystal structures with AChBP. Both *d*-TC and strychnine present with multiple ligand orientations in the LBP, suggesting that these ligands could stabilize the homopentameric protein in an asymmetric state. Additionally, the unconventional state of C-loop induced by the widely used nAChRs inhibitor DHβE proposes a new antagonism mechanism compared to traditional competitive antagonists.

#### The Applications of the Co-crystal Structures

The ultimate goal of the structure-function studies of ligands at nAChRs is to facilitate the design of therapeutic reagents targeting nAChRs implicated in specific diseases. Such applications of nAChRs has been proved all pervasive through the recent and ongoing progress in characterizing the co-crystal structures of ligands with nAChR structural surrogates. One representative application is the use of co-crystal structures in the discovery of new drug leads via virtual screening. In an attempt to design anticobratoxin drug, the α-cbtx active binding site for docking was constructed from the α-cbtx/*Ac*-AChBP crystal complex. Three potential candidates were then selected following the virtual screening of compounds at this site, which can serve as novel templates for the design of promising anticobratoxin drugs (Utsintong et al., 2009).

The co-crystal structures could be a good starting point for the design of novel toxins with improved affinity and desired selectivity. For example, PnIA[A10L D14K]/*Ac*-AChBP structure was used to design a series of PnIA analogs with better affinity for AChBP and α7 nAChRs (Kasheverov et al., 2011). Interestingly, when numerous α-conotoxins were assessed to compete with the iodinated version of the resulting PnIA analog, PnIA[L5R A10L D14R], via a competition binding assay, the IC_50_ of these α-conotoxins were 10-fold lower than those obtained in the competition with the traditional ligand [^125^I]-α-bgtx. It should be noted that although [^125^I]-α-bgtx is currently a popular choice to evaluate the affinity of novel compounds at α7 nAChRs, this toxin has its own weaknesses such as its irreversible binding to target nAChRs (Otvos et al., 2019). As a result, the radio-iodinated PnIA[L5R A10L D14R] could be a more convenient radiolabeled tool in the evaluation of α-conotoxin with potential cholinergic activity compared to [^125^I]- α-bgtx. In addition, the LvIA/*Ac*-AChBP complex has helped to identify key residues in the high preference of LvIA for α3β2 nAChR over α6β2^∗^ nAChR (Xu et al., 2017), which expands our understanding from α-conotoxin MII having high affinity for both α3β2 versus α6β2^∗^ nAChR (Olivera et al., 2008). The identification of α3β2 determinants from LvIA, therefore, could facilitate the designs of optimized α3β2-selective ligands.

The co-crystal structures of these ligands with nAChR structural surrogates have also aided in the design of rationally optimized natural inhibitors that can overcome current challenges in drug development. Such major challenges are the discrepancy in the ligand selectivity at different species of nAChRs (rat versus human), the lack of “drug-like” characteristics and the undesired off-target interactions. The first challenge is mainly seen with α-conotoxins, in which they display lower potency at human nAChR versus rat subtype. This hinders the translation of *in vivo* potentials of toxins into clinical usage. However, a possible explanation for the difference in selectivity among species has been proposed for PeIA with the help of PeIA/*Ac*-AChBP co-crystal structure as described above. This discovery could denote important implications in the development of α-conotoxin PeIA as therapeutic reagents targeting human α6/α3β4 nAChRs (Hone et al., 2013). The second challenge represents the biggest problem in developing toxins into therapeutic reagents. In order to improve MLA “drug-like” characteristic, a series of novel analogs of MLA incorporating either an alcohol or anthranilate ester side chain was designed. The functional results of these analogs when combined with the data from the MLA/*Ac*-AChBP crystal complex allow the role of anthranilate side chain in MLA inhibition at nAChRs to be delaminated (Quek et al., 2010). Another example is the applications of cyclic imine toxins in neurological diseases. Despite their high potency and selectivity, peptidic toxins have not been successfully applied into therapeutic usages, particularly as drugs leads for the treatment of neurological diseases as they are commonly administered parenterally and are unable to pass through the blood-brain barrier due to their polar nature (King, 2011). Meanwhile, imine toxins with their macrocyclic imine framework have shown to traverse the blood-brain barrier as evidenced from the reported presence of SPX in the brain quickly after its intraperitoneal administration to mouse model of Alzheimer’s disease (Veber et al., 2002; Otvos et al., 2019). SPX was also found to be absorbed when administered orally (Otero et al., 2012; Alonso et al., 2013). As a result, compared to peptidic toxins, cyclic imine toxins are promising candidates to be used for neurological diseases. Hence, understanding the interactions between the structural moieties of this new group and AChBP at atomic level from the co-crystal structures could assist with formulating cyclic imine toxins into drug leads. Moreover, the information obtained here could also be applied for peptidic toxins to design analogs suitable for therapeutic applications. The last challenge is often seen with small molecule drugs (Rao et al., 2019). Their small size and simple chemical structures result in its limited interactions at the LBP, thereby its lack of high specificity as evident from the aforementioned small molecule antagonists from plant toxins. As such, despite a number of advantages, the off-target interactions exerted by small molecules drug often lead to drug attrition in pharmaceutical research and development. In contrast, peptidic toxins typically bind with high specificity. Thus, peptide binding interactions underlying their specificity identified from the co-crystal structures, particularly interactions at regions where small molecules and peptide binding site overlap, could guide the optimisation of small molecule antagonists to further improve their performance characteristics.

## Conclusion

Challenges for researchers and clinicians to elucidate the role of particular nAChR subtypes arise from the vast diversity of neuronal nAChRs subtypes expressed in the CNS and PNS. This review aims to give an overview of the utilization of different nAChRs inhibitors from naturally occurring toxins to probe nAChRs. Particularly, the co-crystal structures of these inhibitors with AChBP have aided in the better characterization of the structural mechanism underlying natural toxins potency at different nAChRs subtypes. Information obtained here would be useful in the development of therapeutic reagents targeting nAChRs for the treatment of a wide range of diseases.

## Author Contributions

TH performed bibliography research, wrote the manuscript, and prepared the figures. NA and RL provided scope, guidance, and critically reviewed the manuscript. All authors contributed to the article and approved the submitted version.

## Conflict of Interest

The authors declare that the research was conducted in the absence of any commercial or financial relationships that could be construed as a potential conflict of interest.

## Abbreviations

nAChRs: nicotinic acetylcholine receptors
LBP: ligand binding pocket
PNS: peripheral nervous system
CNS: central nervous system
AChBP: acetylcholine binding protein
*Ls*: *Lymnaea Stagnalis*
*Ac*: *Aplysia californica*
ECD: extracellular domain
TFTs: three-finger toxins
α-cbtx: α-cobratoxin
α-bgtx: α-bungarotoxin
MLA: methyllycaconitine
*d*-TC: *d*-tubocurarine
DH β E: (+)-dihydro-β-erythroidine
SPX: 13-desmethyl spirolide C
GYM: gymonodimines A
PnTx: pinnatoxin
CLR: Cys-loop receptor.

## References

[B1] Abraham N. Healy M. Ragnarsson L. Brust A. Alewood P. F. Lewis R. J. (2017). Structural mechanisms for α-conotoxin activity at the human α3β4 nicotinic acetylcholine receptor. *Sci. Rep.* 7 45466–45466. 10.1038/srep45466 28361878 PMC5374441

[B2] Albuquerque E. X. Pereira E. F. R. Alkondon M. Rogers S. W. (2009). Mammalian nicotinic acetylcholine receptors: from structure to function. *Physiol. Rev.* 89 73–120. 10.1152/physrev.00015.2008 19126755 PMC2713585

[B3] Alonso E. Otero P. Vale C. Alfonso A. Antelo A. Giménez-Llort L. (2013). Benefit of 13-desmethyl spirolide C treatment in triple transgenic mouse model of Alzheimer disease: beta-amyloid and neuronal markers improvement. *Curr. Alzheimer Res.* 10 279–289. 10.2174/1567205011310030007 23036025

[B4] Aráoz R. Ouanounou G. Iorga B. I. Goudet A. Alili D. Amar M. (2015). The neurotoxic effect of 13,19-didesmethyl and 13-desmethyl spirolide C phycotoxins is mainly mediated by nicotinic rather than muscarinic acetylcholine receptors. *Toxicol. Sci.* 147 156–167. 10.1093/toxsci/kfv119 26063663

[B5] Balfour D. J. (2004). The neurobiology of tobacco dependence: a preclinical perspective on the role of the dopamine projections to the nucleus accumbens [corrected]. *Nicotine Tob. Res.* 6 899–912. 10.1080/14622200412331324965 15801566

[B6] Bergmeier S. C. Ismail K. A. Arason K. M. Mckay S. Bryant D. L. Mckay D. B. (2004). Structure activity studies of ring E analogues of methyllycaconitine. Part 2: Synthesis of antagonists to the α3β4^∗^ nicotinic acetylcholine receptors through modifications to the ester. *Bioorg. Med. Chem. Lett.* 14 3739–3742. 10.1016/j.bmcl.2004.05.001 15203153

[B7] Bertrand D. Gopalakrishnan M. (2007). Allosteric modulation of nicotinic acetylcholine receptors. *Biochem. Pharmacol.* 74 1155–1163. 10.1016/j.bcp.2007.07.011 17707779

[B8] Bertrand D. Ballivet M. Rungger D. (1990). Activation and blocking of neuronal nicotinic acetylcholine receptor reconstituted in *Xenopus* oocytes. *Proc. Natl. Acad. Sci. U S A.* 87 1993–1997. 10.1073/pnas.87.5.1993 1968642 PMC53611

[B9] Bertrand D. Devillers-Thiéry A. Revah F. Galzi J. L. Hussy N. Mulle C. (1992). Unconventional pharmacology of a neuronal nicotinic receptor mutated in the channel domain. *Proc. Natl. Acad. Sci. U S A.* 89 1261–1265. 10.1073/pnas.89.4.1261 1741378 PMC48429

[B10] Bisset N. G. (1991). One man’s poison, another man’s medicine? *J. Ethnopharmacol.* 32 71–81. 10.1016/0378-8741(91)90105-m 1881170

[B11] Bourne Y. Radic Z. Araoz R. Talley T. T. Benoit E. Servent D. (2010). Structural determinants in phycotoxins and AChBP conferring high affinity binding and nicotinic AChR antagonism. *Proc. Natl. Acad. Sci. U S A.* 107 6076–6081. 10.1073/pnas.0912372107 20224036 PMC2851920

[B12] Bourne Y. Sulzenbacher G. Radic Z. Araoz R. Reynaud M. Benoit E. (2015). Marine macrocyclic imines, Pinnatoxins A and G: structural determinants and functional properties to distinguish neuronal α7 from muscle α1(2)βγδ nAChRsl. *Structure* 23 1106–1115. 10.1016/j.str.2015.04.009 26004441 PMC4461042

[B13] Bourne Y. Talley T. T. Hansen S. B. Taylor P. Marchot P. (2005). Crystal structure of α-Cbtx–AChBP complex reveals essential interactions between snake α−neurotoxins and nicotinic receptors. *EMBO J.* 24 1512–1522. 10.1038/sj.emboj.7600620 15791209 PMC1142565

[B14] Brams M. Pandya A. Kuzmin D. Van Elk R. Krijnen L. Yakel J. L. (2011). A structural and mutagenic blueprint for molecular recognition of strychnine and d-tubocurarine by different cys-loop receptors. *PLoS Biol.* 9:e1001034. 10.1371/journal.pbio.1001034 21468359 PMC3066128

[B15] Brejc K. Van Dijk W. J. Klaassen R. V. Schuurmans M. Van Der Oost J. Smit A. B. (2001). Crystal structure of an ACh-binding protein reveals the ligand-binding domain of nicotinic receptors. *Nature* 411 269–276. 10.1038/35077011 11357122

[B16] Buisson B. Bertrand D. (2002). Nicotine addiction: the possible role of functional upregulation. *Trends Pharmacol. Sci.* 23 130–136. 10.1016/s0165-6147(00)01979-9 11879680

[B17] Celie P. H. N. Kasheverov I. E. Mordvintsev D. Y. Hogg R. C. Van Nierop P. Van Elk R. (2005). Crystal structure of nicotinic acetylcholine receptor homolog AChBP in complex with an α-conotoxin PnIA variant. *Nat. Struct. Biol.* 12 582–588. 10.1038/nsmb951 15951818

[B18] Celie P. H. Van Rossum-Fikkert S. E. Van Dijk W. J. Brejc K. Smit A. B. Sixma T. K. (2004). Nicotine and carbamylcholine binding to nicotinic acetylcholine receptors as studied in AChBP crystal structures. *Neuron* 41 907–914. 10.1016/S0896-6273(04)00115-1 15046723

[B19] Cembella A. D. Lewis N. I. Quilliam M. A. (1999). Spirolide composition of micro-extracted pooled cells isolated from natural plankton assemblages and from cultures of the dinoflagellate *Alexandrium ostenfeldii*. *Nat. Toxins* 7 197–206. 10.1002/1522-7189(200009/10)7 10945482

[B20] Chang C. C. Lee C. Y. (1963). Isolation of neurotoxins from the venom of *Bungarus multicintus* and their modes of neuromuscular blocking action. *Arch. Int. Pharmacodyn. Ther.* 144 241–257. 14043649

[B21] Chiappinelli V. A. (1983). K-bungarotoxin: a probe for the neuronal nicotinic receptor in the avian ciliary ganglion. *Brain Res.* 277 9–22. 10.1016/0006-8993(83)90902-2 6139146

[B22] Colombo S. F. Mazzo F. Pistillo F. Gotti C. (2013). Biogenesis, trafficking and up-regulation of nicotinic ACh receptors. *Biochem. Pharmacol.* 86 1063–1073. 10.1016/j.bcp.2013.06.023 23830821

[B23] Conti-Fine B. M. Milani M. Kaminski H. J. (2006). Myasthenia gravis: past, present, and future. *J. Clin. Invest.* 116 2843–2854. 10.1172/JCI29894 17080188 PMC1626141

[B24] Corfield P. W. Lee T. J. Low B. W. (1989). The crystal structure of erabutoxin a at 2.0-A resolution. *J. Biol. Chem.* 264 9239–9242. 10.2210/pdb5ebx/pdb 2722828

[B25] Craig A. G. Bandyopadhyay P. Olivera B. M. (1999). Post-translationally modified neuropeptides from *Conus* venoms. *Eur. J. Biochem.* 264 271–275. 10.1046/j.1432-1327.1999.00624 10491070

[B26] Daly J. W. (2005). Nicotinic agonists, antagonists, and modulators from natural sources. *Cell. Mol. Neurobiol.* 25 513–552. 10.1007/s10571-005-3968-4 16075378 PMC11529529

[B27] D’Andrea M. R. Nagele R. G. (2006). Targeting the alpha 7 nicotinic acetylcholine receptor to reduce amyloid accumulation in Alzheimer’s disease pyramidal neurons. *Curr. Pharm. Des.* 12 677–684. 10.2174/138161206775474224 16472157

[B28] Dani J. A. Bertrand D. (2007). Nicotinic acetylcholine receptors and nicotinic cholinergic mechanisms of the central nervous system. *Annu. Rev. Pharmacol. Toxicol.* 47 699–729. 10.1146/annurev.pharmtox.47.120505.105214 17009926

[B29] De Biasi M. Dani J. A. (2011). Reward, addiction, withdrawal to nicotine. *Annu. Rev. Neurosci.* 34 105–130. 10.1146/annurev-neuro-061010-113734 21438686 PMC3137256

[B30] Dellisanti C. D. Yao Y. Stroud J. C. Wang Z.-Z. Chen L. (2007). Crystal structure of the extracellular domain of nAChR α1 bound to α-bungarotoxin at 1.94 Å resolution. *Nat. Neurosci.* 10 953–962. 10.1038/nn1942 17643119

[B31] Dutertre S. Nicke A. Lewis R. J. (2005). β2 subunit contribution to 4/7 alpha-conotoxin binding to the nicotinic acetylcholine receptor. *J. Biol. Chem.* 280 30460–30468. 10.1074/jbc.M504229200 15929983

[B32] Dutertre S. Nicke A. Tsetlin V. I. (2017). Nicotinic acetylcholine receptor inhibitors derived from snake and snail venoms. *Neuropharmacology* 127 196–223. 10.1016/j.neuropharm.2017.06.011 28623170

[B33] Dutertre S. Nicke A. Tyndall J. D. A. Lewis R. J. (2004). Determination of α-conotoxin binding modes on neuronal nicotinic acetylcholine receptors. *J. Mol. Recognit.* 17 339–347. 10.1002/jmr.683 15227641

[B34] Dutertre S. Ulens C. Buttner R. Fish A. Van Elk R. Kendel Y. (2007). AChBP-targeted α-conotoxin correlates distinct binding orientations with nAChR subtype selectivity. *EMBO J.* 26 3858–3867. 10.1038/sj.emboj.7601785 17660751 PMC1952216

[B35] Ellison M. Feng Z.-P. Park A. J. Zhang X. Olivera B. M. McIntosh J. M. (2008). α-RgIA, a novel conotoxin that blocks the α9α10 nAChR: structure and identification of key receptor-binding residues. *J. Mol. Biol.* 377 1216–1227. 10.1016/j.jmb.2008.01.082 18295795 PMC2376044

[B36] Ellison M. Haberlandt C. Gomez-Casati M. E. Watkins M. Elgoyhen A. B. McIntosh J. M. (2006). α-RgIA: A novel conotoxin that specifically and potently blocks the α9α10 nAChR. *Biochemistry* 45 1511–1517. 10.1021/bi0520129 16445293

[B37] Engel A. G. Shen X.-M. Selcen D. Sine S. M. (2015). Congenital myasthenic syndromes: pathogenesis, diagnosis, and treatment. *Lancet Neurol.* 14 420–434. 10.1016/S1474-4422(14)70201-7 25792100 PMC4520251

[B38] Fainzilber M. Hasson A. Oren R. Burlingame A. L. Gordon D. Spira M. E. (1994). New mollusc-specific α-Conotoxins block *Aplysia* neuronal acetylcholine receptors. *Biochemistry* 33 9523–9529. 10.1021/bi00198a018 8068627

[B39] Folkers K. Major R. T. (1937). Isolation of Erythrodine, an alkaloid of curare action, from *Erythrina americana mill*. *J. Am. Chem. Soc.* 59 1580–1581. 10.1021/ja01287a509

[B40] Freedman R. Hall M. Adler L. E. Leonard S. (1995). Evidence in postmortem brain tissue for decreased numbers of hippocampal nicotinic receptors in schizophrenia. *Biol. Psychiatry* 38 22–33. 10.1016/0006-3223(94)00252-x 7548469

[B41] Fruchart-Gaillard C. Gilquin B. Antil-Delbeke S. Le Novère N. Tamiya T. Corringer P.-J. (2002). Experimentally based model of a complex between a snake toxin and the α7 nicotinic receptor. *Proc. Natl. Acad. Sci. U S A.* 99:3216. 10.1073/pnas.042699899 11867717 PMC122499

[B42] Fry B. G. Vidal N. Norman J. A. Vonk F. J. Scheib H. Ramjan S. F. R. (2006). Early evolution of the venom system in lizards and snakes. *Nature* 439 584–588. 10.1038/nature04328 16292255

[B43] Giniatullin R. Nistri A. Yakel J. L. (2005). Desensitization of nicotinic ACh receptors: shaping cholinergic signaling. *Trends Neurosci.* 28 371–378. 10.1016/j.tins.2005.04.009 15979501

[B44] Gotti C. Clementi F. (2004). Neuronal nicotinic receptors: from structure to pathology. *Prog. Neurobiol.* 74 363–396. 10.1016/j.pneurobio.2004.09.006 15649582

[B45] Green B. T. Welch K. D. Panter K. E. Lee S. T. (2013). Plant toxins that affect nicotinic acetylcholine receptors: a review. *Chem. Res. Toxicol.* 26 1129–1138. 10.1021/tx400166f 23848825

[B46] Grudzinska J. Schemm R. Haeger S. Nicke A. Schmalzing G. Betz H. (2005). The β subunit determines the ligand binding properties of synaptic glycine receptors. *Neuron* 45 727–739. 10.1016/j.neuron.2005.01.028 15748848

[B47] Hansen S. B. Radić Z. Talley T. T. Molles B. E. Deerinck T. Tsigelny I. (2002). Tryptophan fluorescence reveals conformational changes in the acetylcholine binding protein. *J. Biol. Chem.* 277 41299–41302. 10.1074/jbc.C200462200 12235129 PMC3191908

[B48] Hansen S. B. Sulzenbacher G. Huxford T. Marchot P. Taylor P. Bourne Y. (2005). Structures of *Aplysia* AChBP complexes with nicotinic agonists and antagonists reveal distinctive binding interfaces and conformations. *EMBO J.* 24 3635–3646. 10.1038/sj.emboj.7600828 16193063 PMC1276711

[B49] Hansen S. B. Talley T. T. Radic Z. Taylor P. (2004). Structural and ligand recognition characteristics of an acetylcholine-binding protein from *Aplysia californica*. *J. Biol. Chem.* 279 24197–24202. 10.1074/jbc.M402452200 15069068 PMC4762456

[B50] Harvey S. C. Luetje C. W. (1996). Determinants of competitive antagonist sensitivity on neuronal nicotinic receptor β subunits. *J. Neurosci.* 16 3798–3806. 10.1523/JNEUROSCI.16-12-03798.1996 8656274 PMC6578601

[B51] Hauser T. A. Hepler C. D. Kombo D. C. Grinevich V. P. Kiser M. N. Hooker D. N. (2012). Comparison of acetylcholine receptor interactions of the marine toxins, 13-desmethylspirolide C and gymnodimine. *Neuropharmacology* 62 2239–2250. 10.1016/j.neuropharm.2012.01.009 22306792

[B52] Haywood A. J. Steidinger K. A. Truby E. W. Bergquist P. R. Bergquist P. L. Adamson J. (2004). Comparative morphology and molecular phylogenetic analysis of three new species of the genus *Karenia (dinophycea)* from New Zealand. *J. Phycol.* 40 165–179. 10.1111/j.0022-3646.2004.02-149.x

[B53] Hellyer S. D. Selwood A. I. Van Ginkel R. Munday R. Sheard P. Miles C. O. (2014). *In vitro* labelling of muscle type nicotinic receptors using a fluorophore-conjugated pinnatoxin F derivative. *Toxicon* 87 17–25. 10.1016/j.toxicon.2014.05.013 24887283

[B54] Hogg R. C. Bertrand D. (2004). Nicotinic acetylcholine receptors as drug targets. *Curr. Drug. Targets CNS Neurol. Disord.* 3 123–130. 10.2174/1568007043482507 15078187

[B55] Hogg R. C. Hopping G. Alewood P. F. Adams D. J. Bertrand D. (2003a). α-conotoxins PnIA and [A10L]PnIA stabilize different states of the α7-L247T nicotinic acetylcholine receptor. *J. Biol. Chem.* 278 26908–26914. 10.1074/jbc.M212628200 12746432

[B56] Hogg R. C. Miranda L. P. Craik D. J. Lewis R. J. Alewood P. F. Adams D. J. (1999). Single amino acid substitutions in α-conotoxin PnIA shift selectivity for subtypes of the mammalian neuronal nicotinic acetylcholine receptor. *J. Biol. Chem.* 274 36559–36564. 10.1074/jbc.274.51.36559 10593955

[B57] Hogg R. C. Raggenbass M. Bertrand D. (2003b). Nicotinic acetylcholine receptors: from structure to brain function. *Rev. Physiol. Biochem. Pharmacol.* 147 1–46. 10.1007/s10254-003-0005-1 12783266

[B58] Hone A. J. McIntosh J. M. (2018). Nicotinic acetylcholine receptors in neuropathic and inflammatory pain. *FEBS Lett.* 592 1045–1062. 10.1002/1873-3468.12884 29030971 PMC5899685

[B59] Hone A. J. Ruiz M. Scadden M. Christensen S. Gajewiak J. Azam L. (2013). Positional scanning mutagenesis of α-conotoxin PeIA identifies critical residues that confer potency and selectivity for α6/α3β2β3 and α3β2 nicotinic acetylcholine receptors. *J. Biol. Chem.* 288 25428–25439. 10.1074/jbc.M113.482059 23846688 PMC3757205

[B60] Hone A. J. Servent D. McIntosh J. M. (2018a). α9-containing nicotinic acetylcholine receptors and the modulation of pain. *Br. J. Pharmacol.* 175 1915–1927. 10.1111/bph.13931 28662295 PMC5980226

[B61] Hone A. J. Talley T. T. Bobango J. Huidobro Melo C. Hararah F. Gajewiak J. (2018b). Molecular determinants of α-conotoxin potency for inhibition of human and rat α6β4 nicotinic acetylcholine receptors. *J Biol. Chem.* 293 17838–17852. 10.1074/jbc.RA118.005649 30249616 PMC6240866

[B62] Hope A. G. Belelli D. Mair I. D. Lambert J. J. Peters J. A. (1999). Molecular determinants of (+)-Tubocurarine binding at recombinant 5-Hydroxytryptamine receptor subunits. *Mol. Pharmacol.* 55:1037. 10.1124/mol.55.6.1037 10347245

[B63] Hu S.-H. Gehrmann J. W Guddat L. Alewood P. F. Craik D. J. Martin J. L. (1996). The 1.1 Å crystal structure of the neuronal acetylcholine receptor antagonist, α-conotoxin PnIA from *Conus pennaceus*. *Structure* 4 417–423. 10.1016/S0969-2126(96)00047-0 8740364

[B64] Hu T. Burton I. W. Cembella A. D. Curtis J. M. Quilliam M. A. Walter J. A. (2001). Characterization of spirolides A, C, and 13-desmethyl C, new marine toxins isolated from toxic plankton and contaminated shellfish. *J. Nat. Prod.* 64 308–312. 10.1021/np000416q 11277745

[B65] Huang S. Li S. X. Bren N. Cheng K. Gomoto R. Chen L. (2013). Complex between α-bungarotoxin and an α7 nicotinic receptor ligand-binding domain chimaera. *Biochem. J.* 454 303–310. 10.1042/BJ20130636 23800261 PMC3920732

[B66] Hurst R. Rollema H. Bertrand D. (2013). Nicotinic acetylcholine receptors: From basic science to therapeutics. *Pharmacol. Ther.* 137 22–54. 10.1016/j.pharmthera.2012.08.012 22925690

[B67] Hussain G. Rasul A. Anwar H. Aziz N. Razzaq A. Wei W. (2018). Role of plant derived alkaloids and their mechanism in neurodegenerative disorders. *Int. J. Biol. Sci.* 14 341–357. 10.7150/ijbs.23247 29559851 PMC5859479

[B68] Inserra M. C. Kompella S. N. Vetter I. Brust A. Daly N. L. Cuny H. (2013). Isolation and characterization of α-conotoxin LsIA with potent activity at nicotinic acetylcholine receptors. *Biochem. Pharmacol.* 86 791–799. 10.1016/j.bcp.2013.07.016 23924607

[B69] Iturriaga-Vasquez P. Carbone A. Garcia-Beltran O. Livingstone P. D. Biggin P. C. Cassels B. K. (2010). Molecular determinants for competitive inhibition of α4β2 nicotinic acetylcholine receptors. *Mol. Pharmacol.* 78 366–375. 10.1124/mol.110.065490 20547737 PMC2939478

[B70] Janes R. W. (2005). α-Conotoxins as selective probes for nicotinic acetylcholine receptor subclasses. *Curr. Opin. Pharmacol.* 5 280–292. 10.1016/j.coph.2005.01.013 15907916

[B71] Jennings K. R. Brown D. G. Wright D. P. (1986). Methyllycaconitine, a naturally occurring insecticide with a high affinity for the insect cholinergic receptor. *Experientia* 42 611–613. 10.1007/bf01955557

[B72] Jensen A. A. Frolund B. Liljefors T. Krogsgaard-Larsen P. (2005). Neuronal nicotinic acetylcholine receptors: structural revelations, target identifications, and therapeutic inspirations. *J. Med. Chem.* 48 4705–4745. 10.1021/jm040219e 16033252

[B73] Jepsen T. H. Jensen A. A. Lund M. H. Glibstrup E. Kristensen J. L. (2014). Synthesis and pharmacological evaluation of DHβE analogues as neuronal nicotinic acetylcholine receptor antagonists. *ACS Med. Chem. Lett.* 5 766–770. 10.1021/ml500094c 25050162 PMC4094251

[B74] Johnson J. W. Ascher P. (1987). Glycine potentiates the NMDA response in cultured mouse brain neurons. *Nature* 325 529–531. 10.1038/325529a0 2433595

[B75] Karlsson E. Heilbronn E. Widlund L. (1972). Isolation of the nicotinic acetylcholine receptor by biospecific chromatography on insolubilized *Naja naja* neurotoxin. *FEBS Lett.* 28 107–111. 10.1016/0014-5793(72)80688-4 4646866

[B76] Kasheverov I. E. Zhmak M. N. Khruschov A. Y. Tsetlin V. I. (2011). Design of new α-conotoxins: from computer modeling to synthesis of potent cholinergic compounds. *Mar. drugs* 9 1698–1714. 10.3390/md9101698 22072993 PMC3210602

[B77] Kessler P. Marchot P. Silva M. Servent D. (2017). The three-finger toxin fold: a multifunctional structural scaffold able to modulate cholinergic functions. *J. Neurochem.* 142 7–18. 10.1111/jnc.13975 28326549

[B78] King G. F. (2011). Venoms as a platform for human drugs: translating toxins into therapeutics. *Expert Opin. Biol. Ther.* 11 1469–1484. 10.1517/14712598.2011.621940 21939428

[B79] Kouvatsos N. Giastas P. Chroni-Tzartou D. Poulopoulou C. Tzartos S. J. (2016). Crystal structure of a human neuronal nAChR extracellular domain in pentameric assembly: Ligand-bound α2 homopentamer. *Proc. Natl. Acad. Sci. U S A.* 113:9635. 10.1073/pnas.1602619113 27493220 PMC5003238

[B80] Kukel C. F. Jennings K. R. (1994). *Delphinium* alkaloids as inhibitors of α-Bungarotoxin binding to rat and insect neural membranes. *Can. J. Physiol. Pharmacol.* 72 104–107. 10.1139/y94-016 8012891

[B81] Lebbe E. K. M. Peigneur S. Wijesekara I. Tytgat J. (2014). Conotoxins targeting nicotinic acetylcholine receptors: an overview. *Mar. Drugs* 12 2970–3004. 10.3390/md12052970 24857959 PMC4052327

[B82] Lewis R. J. Garcia M. L. (2003). Therapeutic potential of venom peptides. *Nat. Rev. Drug. Discov.* 2:790. 10.1038/nrd1197 14526382

[B83] Lewis R. J. Dutertre S. Vetter I. Christie M. J. (2012). Conus venom peptide pharmacology. *Pharmacol. Rev.* 64 259–298. 10.1124/pr.111.005322 22407615

[B84] Li S.-X. Huang S. Bren N. Noridomi K. Dellisanti C. D. Sine S. M. (2011). Ligand-binding domain of an α7-nicotinic receptor chimera and its complex with agonist. *Nat. Neurosci.* 14 1253–1259. 10.1038/nn.2908 21909087 PMC3489043

[B85] Lin B. Xu M. Zhu X. Wu Y. Liu X. Zhangsun D. (2016). From crystal structure of α-conotoxin GIC in complex with *Ac*-AChBP to molecular determinants of its high selectivity for α3β2 nAChR. *Sci. Rep.* 6:22349. 10.1038/srep22349 26925840 PMC4772116

[B86] Lindstrom J. (1997). Nicotinic acetylcholine receptors in health and disease. *Mol. Neurobiol.* 15 193–222. 10.1007/bf02740634 9396010

[B87] Liu X. Xu Y. Li H. Wang X. Jiang H. Barrantes F. J. (2008). Mechanics of channel gating of the nicotinic acetylcholine receptor. *PLoS Comput. Biol.* 4:e19. 10.1371/journal.pcbi.0040019 18225945 PMC2211534

[B88] Luo S. Nguyen T. A. Cartier G. E. Olivera B. M. Yoshikami D. McIntosh J. M. (1999). Single-residue alteration in α-conotoxin PnIA switches its nAChR subtype selectivity. *Biochemistry* 38 14542–14548. 10.1021/bi991252j 10545176

[B89] Luo S. Zhangsun D. Schroeder C. I. Zhu X. Hu Y. Wu Y. (2014). A novel α4/7-conotoxin LvIA from *Conus lividus* that selectively blocks α3β2 vs. α6/α3bβ2β3 nicotinic acetylcholine receptors. *Faseb J.* 28 1842–1853. 10.1096/fj.13-244103 24398291 PMC3963015

[B90] Majinda R. R. T. (2018). An update of *Erythrinan* alkaloids and their pharmacological activities. *Prog. Chem. Org. Nat. Prod.* 107 95–159. 10.1007/978-3-319-93506-5_2 30178271

[B91] Maslennikov I. V. Shenkarev Z. O. Zhmak M. N. Ivanov V. T. Methfessel C. Tsetlin V. I. (1999). NMR spatial structure of α-conotoxin ImI reveals a common scaffold in snail and snake toxins recognizing neuronal nicotinic acetylcholine receptors. *FEBS Lett.* 444 275–280. 10.1016/s0014-5793(99)00069-1 10050774

[B92] Matsubayashi H. Alkondon M. Pereira E. F. R. Swanson K. L. Albuquerque E. X. (1998). Strychnine: a potent competitive antagonist of α-Bungarotoxin-Sensitive nicotinic acetylcholine receptors in rat hippocampal neurons. *J. Pharmacol. Exp. Ther.* 284:904. 9495848

[B93] McIntosh J. M. Dowell C. Watkins M. Garrett J. E. Yoshikami D. Olivera B. M. (2002). α-Conotoxin GIC from *Conus geographus*, a novel peptide antagonist of nicotinic acetylcholine receptors. *J. Biol. Chem.* 277 33610–33615. 10.1074/jbc.M205102200 12114524

[B94] McIntosh J. M. Olivera B. M. Cruz L. J. (1999a). Conus peptides as probes for ion channels. *Methods Enzymol.* 294 605–624. 10.1016/S0076-6879(99)94034-X 9916250

[B95] McIntosh J. M. Santos A. D. Olivera B. M. (1999b). Conus peptides targeted to specific nicotinic acetylcholine receptor subtypes. *Annu. Rev. Biochem.* 68 59–88. 10.1146/annurev.biochem.68.1.59 10872444

[B96] McIntosh J. M. Yoshikami D. Mahe E. Nielsen D. B. Rivier J. E. Gray W. R. (1994). A nicotinic acetylcholine receptor ligand of unique specificity, α-conotoxin ImI. *J. Biol. Chem.* 269 16733–16739. 8206995

[B97] McLane K. E. Weaver W. R. Lei S. Chiappinelli V. A. Conti-Tronconi B. M. (1993). Homologous κ-neurotoxins exhibit residue-specific interactions with the α3 subunit of the nicotinic acetylcholine receptor: A comparison of the structural requirements for κ-bungarotoxin and κ-flavitoxin binding. *Biochemistry* 32 6988–6994. 10.1021/bi00078a025 8334127

[B98] Millar N. S. Harkness P. C. (2008). Assembly and trafficking of nicotinic acetylcholine receptors (Review). *Mol. Membr. Biol.* 25 279–292. 10.1080/09687680802035675 18446614

[B99] Moise L. Piserchio A. Basus V. J. Hawrot E. (2002). NMR structural analysis of α-bungarotoxin and its complex with the principal α-neurotoxin-binding sequence on the alpha 7 subunit of a neuronal nicotinic acetylcholine receptor. *J. Biol. Chem.* 277 12406–12417. 10.1074/jbc.M110320200 11790782

[B100] Molgó J. Marchot P. Aráoz R. Benoit E. Iorga B. I. Zakarian A. (2017). Cyclic imine toxins from dinoflagellates: a growing family of potent antagonists of the nicotinic acetylcholine receptors. *J. Neurochem.* 142(Suppl. 2), 41–51. 10.1111/jnc.13995 28326551 PMC5550345

[B101] Munday R. Quilliam M. A. Leblanc P. Lewis N. Gallant P. Sperker S. A. (2012). Investigations into the toxicology of spirolides, a group of marine phycotoxins. *Toxins* 4 1–14. 10.3390/toxins4010001 22347619 PMC3277094

[B102] Nemecz A. Taylor P. (2011). Creating an α7 nicotinic acetylcholine recognition domain from the acetylcholine-binding protein: crystallographic and ligand selectivity analyses. *J. Biol. Chem.* 286 42555–42565. 10.1074/jbc.M111.286583 22009746 PMC3234955

[B103] Nicke A. Loughnan M. L. Millard E. L. Alewood P. F. Adams D. J. Daly N. L. (2003). Isolation, structure, and activity of GID, a novel α4/7-conotoxin with an extended N-terminal sequence. *J. Biol. Chem.* 278 3137–3144. 10.1074/jbc.M210280200 12419800

[B104] Nicke A. Wonnacott S. Lewis R. J. (2004). α-conotoxins as tools for the elucidation of structure and function of neuronal nicotinic acetylcholine receptor subtypes. *Eur. J. Biochem.* 271 2305–2319. 10.1111/j.1432-1033.2004.04145.x 15182346

[B105] Olivera B. M. Quik M. Vincler M. McIntosh J. M. (2008). Subtype-selective conopeptides targeted to nicotinic receptors: Concerted discovery and biomedical applications. *Channels* 2 143–152. 10.4161/chan.2.2.6276 18849660

[B106] Olsen J. A. Balle T. Gajhede M. Ahring P. K. Kastrup J. S. (2014). Molecular recognition of the neurotransmitter acetylcholine by an acetylcholine binding protein reveals determinants of binding to nicotinic acetylcholine receptors. *PLoS One* 9:e91232. 10.1371/journal.pone.0091232 24637639 PMC3956608

[B107] Olsen R. W. Meunier J.-C. Changeux J.-P. (1972). Progress in the purification of the cholinergic receptor protein from *Electrophorus electricus* by affinity chromatography. *FEBS Lett.* 28 96–100. 10.1016/0014-5793(72)80686-0 4646881

[B108] Otero A. Chapela M.-J. Atanassova M. Vieites J. M. Cabado A. G. (2011). Cyclic imines: chemistry and mechanism of action: a review. *Chem. Res. Toxicol.* 24 1817–1829. 10.1021/tx200182m 21739960

[B109] Otero P. Alfonso A. Rodríguez P. Rubiolo J. A. Cifuentes J. M. Bermúdez R. (2012). Pharmacokinetic and toxicological data of spirolides after oral and intraperitoneal administration. *Food Chem. Toxicol.* 50 232–237. 10.1016/j.fct.2011.10.062 22100396

[B110] Otvos R. A. Still K. B. M. Somsen G. W. Smit A. B. Kool J. (2019). Drug discovery on natural products: from ion channels to nAChRs, from nature to libraries, from analytics to assays. *SLAS Discov.* 24 362–385. 10.1177/2472555218822098 30682257 PMC6484542

[B111] Palma E. Bertrand S. Binzoni T. Bertrand D. (1996). Neuronal nicotinic α7 receptor expressed in *Xenopus* oocytes presents five putative binding sites for methyllycaconitine. *J. Physiol.* 491(Pt 1), 151–161. 10.1113/jphysiol.1996.sp021203 9011607 PMC1158766

[B112] Papineni R. V. Pedersen S. E. (1997). Interaction of *d*-tubocurarine analogs with the mouse nicotinic acetylcholine receptor. Ligand orientation at the binding site. *J. Biol. Chem.* 272 24891–24898. 10.1074/jbc.272.40.24891 9312090

[B113] Picot C. Nguyen T. A. Roudot A. C. Parent-Massin D. (2011). A preliminary risk assessment ofhuman exposure to phycotoxins in shellfish: a review. *Hum. Ecol. Risk Assess.* 17 328–366. 10.1080/10807039.2011.552393

[B114] Prashanth J. R. Lewis R. J. Dutertre S. (2012). Towards an integrated venomics approach for accelerated conopeptide discovery. *Toxicon* 60 470–477. 10.1016/j.toxicon.2012.04.340 22564717

[B115] Quek G. X. J. Lin D. Halliday J. I. Absalom N. Ambrus J. I. Thompson A. J. (2010). Identifying the binding site of novel methyllycaconitine (MLA) analogs at α4β2 nicotinic acetylcholine receptors. *ACS Chem. Neurosci.* 1 796–809. 10.1021/cn100073x 22778816 PMC3368652

[B116] Quiram P. A. Jones J. J. Sine S. M. (1999). Pairwise interactions between neuronal α7 acetylcholine receptors and α-conotoxin ImI. *J. Biol. Chem.* 274 19517–19524. 10.1074/jbc.275.7.4889 10391883

[B117] Ramilo C. A. Zafaralla G. C. Nadasdi L. Hammerland L. G. Yoshikami D. Gray W. R. (1992). Novel α- and ω-conotoxins and Co*nus striatus ve*nom. *Biochemistry* 31 9919–9926. 10.1021/bi00156a009 1390774

[B118] Rao M. S. Gupta R. Liguori M. J. Hu M. Huang X. Mantena S. R. (2019). Novel computational approach to predict off-target interactions for small molecules. *Front. Big Data* 2:25 10.3389/fdata.2019.00025 PMC7931946 33693348

[B119] Ren J. Zhu X. Xu P. Li R. Fu Y. Dong S. (2019). d-Amino acid substitution of α-conotoxin RgIA identifies its critical residues and improves the enzymatic stability. *Mar. drugs* 17:142. 10.3390/md17030142 30823399 PMC6472032

[B120] Rivera H. L. Barrueto F. (2014). “Strychnine,” in *Encyclopedia of Toxicology (Third Edition)*, ed. Wexler P. (Oxford: Academic Press), 407–408.

[B121] Rogers J. P. Luginbühl P. Pemberton K. Harty P. Wemmer D. E. Stevens R. C. (2000). Structure-activity relationships in a peptidic α7 nicotinic acetylcholine receptor antagonist. *J. Mol. Biol.* 304 911–926. 10.1006/jmbi.2000.4247 11124036

[B122] Rucktooa P. Smit A. B. Sixma T. K. (2009). Insight in nAChR subtype selectivity from AChBP crystal structures. *Biochem. Pharmacol.* 78 777–787. 10.1016/j.bcp.2009.06.098 19576182

[B123] Rujjanawate C. Kanjanapothi D. Panthong A. (2003). Pharmacological effect and toxicity of alkaloids from *Gelsemium elegans Benth*. *J. Ethnopharmacol.* 89 91–95. 10.1016/s0378-8741(03)00267-8 14522437

[B124] Satkunanathan N. Livett B. Gayler K. Sandall D. Down J. Khalil Z. (2005). α-conotoxin Vc1.1 alleviates neuropathic pain and accelerates functional recovery of injured neurones. *Brain Res.* 1059 149–158. 10.1016/j.brainres.2005.08.009 16182258

[B125] Sciamanna M. A. Griesmann G. E. Williams C. L. Lennon V. A. (1997). Nicotinic acetylcholine receptors of muscle and neuronal α7 types coexpressed in a small cell lung carcinoma. *J. Neurochem.* 69 2302–2311. 10.1046/j.1471-4159.1997.69062302.x 9375661

[B126] Shahsavar A. Kastrup J. S. Nielsen E. Ø Kristensen J. L. Gajhede M. Balle T. (2012). Crystal structure of *Lymnaea stagnalis* AChBP complexed with the potent nAChR antagonist DHβE suggests a unique mode of antagonism. *PloS One* 7:e40757. 10.1371/journal.pone.0040757 22927902 PMC3425559

[B127] Sine S. M. (2012). End-plate acetylcholine receptor: structure, mechanism, pharmacology, and disease. *Physiol. Rev.* 92 1189–1234. 10.1152/physrev.00015.2011 22811427 PMC3489064

[B128] Sine S. M. Huang S. Li S. X. Dacosta C. J. Chen L. (2013). Inter-residue coupling contributes to high-affinity subtype-selective binding of α-Bungarotoxin to nicotinic receptors. *Biochem J.* 454 311–321. 10.1042/BJ20130638 23802200 PMC3912756

[B129] Smit A. B. Syed N. I. Schaap D. Van Minnen J. Klumperman J. Kits K. S. (2001). A glia-derived acetylcholine-binding protein that modulates synaptic transmission. *Nature* 411 261–268. 10.1038/35077000 11357121

[B130] Steinbach J. H. Sine S. M. (1987). Function of nicotinic acetylcholine receptors. *Soc. Gen. Physiol. Ser.* 41 19–42. 2436310

[B131] Stivala C. E. Benoit E. Aráoz R. Servent D. Novikov A. Molgó J. (2015). Synthesis and biology of cyclic imine toxins, an emerging class of potent, globally distributed marine toxins. *Nat. Prod. Rep.* 32 411–435. 10.1039/c4np00089g 25338021 PMC4344380

[B132] Talcott P. A. (2013). “Chapter 81 - Strychnine,” in *Small Animal Toxicology (Third Edition)*, eds Peterson M. E. Talcott P. A. (Saint Louis: W.B. Saunders), 827–831.

[B133] Tsetlin V. Hucho F. (2004). Snake and snail toxins acting on nicotinic acetylcholine receptors: Fundamental aspects and medical applications. *FEBS Lett.* 557 9–13. 10.1016/S0014-5793(03)01454-6 14741333

[B134] Tuba Z. Maho S. Vizi E. S. (2002). Synthesis and structure-activity relationships of neuromuscular blocking agents. *Curr. Med. Chem.* 9 1507–1536. 10.2174/0929867023369466 12171561

[B135] Ulens C. Hogg R. C. Celie P. H. Bertrand D. Tsetlin V. Smit A. B. (2006). Structural determinants of selective α-conotoxin binding to a nicotinic acetylcholine receptor homolog AChBP. *Proc. Natl. Acad. Sci. U S A.* 103:3615. 10.1073/pnas.0507889103 16505382 PMC1450132

[B136] Unwin N. (1993). Nicotinic acetylcholine receptor at 9 A resolution. *J. Mol. Biol.* 229 1101–1124. 8445638 10.1006/jmbi.1993.1107

[B137] Unwin N. (2005). Refined structure of the nicotinic acetylcholine receptor at 4A resolution. *J. Mol. Biol.* 346 967–989. 10.1016/j.jmb.2004.12.031 15701510

[B138] Utkin Y. N. (2013). Three-finger toxins, a deadly weapon of *elapid* venom–milestones of discovery. *Toxicon* 62 50–55. 10.1016/j.toxicon.2012.09.007 23000250

[B139] Utsintong M. Talley T. T. Taylor P. W. Olson A. J. Vajragupta O. (2009). Virtual screening against α-cobratoxin. *J. Biomol. Screen.* 14 1109–1118. 10.1177/1087057109344617 19734437 PMC3191909

[B140] Veber D. F. Johnson S. R. Cheng H.-Y. Smith B. R. Ward K. W. Kopple K. D. (2002). Molecular properties that influence the oral bioavailability of drug candidates. *J. Med. Chem* 45 2615–2623. 10.1021/jm020017n 12036371

[B141] Wang H. Y. Lee D. H. Davis C. B. Shank R. P. (2000). Amyloid peptide Aβ(1-42) binds selectively and with picomolar affinity to α7 nicotinic acetylcholine receptors. *J. Neurochem.* 75 1155–1161. 10.1046/j.1471-4159.2000.0751155.x 10936198

[B142] Ward J. M. Cockcroft V. B. Lunt G. G. Smillie F. S. Wonnacott S. (1990). Methyllycaconitine: a selective probe for neuronal α-bungarotoxin binding sites. *FEBS Lett.* 270 45–48. 10.1016/0014-5793(90)81231-c 2226787

[B143] Wieskopf J. S. Mathur J. Limapichat W. Post M. R. Al-Qazzaz M. Sorge R. E. (2015). The nicotinic α6 subunit gene determines variability in chronic pain sensitivity via cross-inhibition of P2X2/3 receptors. *Sci. Transl. Med.* 7:287ra272. 10.1126/scitranslmed.3009986 25972004 PMC5018401

[B144] Wintersteiner O. Dutcher J. D. (1943). Curare alkaloids from *Chondodendron tometosum*. *Science* 97 467. 10.1126/science.97.2525.467 17789877

[B145] Xu M. Zhu X. Yu J. Yu J. Luo S. Wang X. (2017). The crystal structure of *Ac*-AChBP in complex with α-conotoxin LvIA reveals the mechanism of its selectivity towards different nAChR subtypes. *Protein Cell* 8 675–685. 10.1007/s13238-017-0426-2 28585176 PMC5563285

[B146] Yakel J. L. Jackson M. B. (1988). 5-HT3 receptors mediate rapid responses in cultured hippocampus and a clonal cell line. *Neuron* 1 615–621. 10.1016/0896-6273(88)90111-0 3272181

[B147] Yan D. Pedersen S. E. White M. M. (1998). Interaction of *d-*tubocurarine analogs with the 5-HT3 receptor. *Neuropharmacology* 37 251–257. 10.1016/s0028-3908(98)00010-0 9680250

[B148] Yum L. Wolf K. M. Chiappinelli V. A. (1996). Nicotinic acetylcholine receptors in separate brain regions exhibit different affinities for methyllycaconitine. *Neuroscience* 72 545–555. 10.1016/0306-4522(95)00531-5 8737423

[B149] Zhang Y. (2015). Why do we study animal toxins? *Dongwuxue Yanjiu* 36 183–222. 10.13918/j.issn.2095-8137.2015.4.183 26228472 PMC4790257

[B150] Zouridakis M. Giastas P. Zarkadas E. Chroni-Tzartou D. Bregestovski P. Tzartos S. J. (2014). Crystal structures of free and antagonist-bound states of human α9 nicotinic receptor extracellular domain. *Nat. Struct. Mol. Biol.* 21 976–980. 10.1038/nsmb.2900 25282151

[B151] Zouridakis M. Papakyriakou A. Ivanov I. A. Kasheverov I. E. Tsetlin V. Tzartos S. (2019). Crystal structure of the monomeric extracellular domain of α9 nicotinic receptor subunit in complex with α-Conotoxin RgIA: molecular dynamics insights into RgIA binding to α9α10 nicotinic receptors. *Front. Pharmacol.* 10:474. 10.3389/fphar.2019.00474 31118896 PMC6504684

